# A multicriteria decision analysis for selecting rainwater harvesting systems in rural areas: a tool for developing countries

**DOI:** 10.1007/s11356-024-33734-8

**Published:** 2024-06-14

**Authors:** Diana Prieto-Jiménez, Edgar Ricardo Oviedo-Ocaña, Sully Gómez-Isidro, Isabel Cristina Domínguez

**Affiliations:** Escuela de Ingeniería Civil, Facultad de Ingenierías Físico-Mecánicas, Universidad Industrial de Santander, Carrera 27 Calle 9, Bucaramanga, Colombia

**Keywords:** Rainwater harvesting, Sustainability, Multicriteria analysis, Multiple uses of water, Rural areas

## Abstract

Global water provision challenges have promoted decentralized water supply alternatives such as rainwater harvesting systems (RWHS). RWHS sustainability demands involve social, technical, and economic criteria in planning. Generally, in rural areas, water provision is more complex due to multiple uses of water, scattering of households, and low economies of scale. This research proposes a multicriteria tool for selecting RWHS in rural areas, considering social, technical, and economic criteria. The tool was developed by systematically identifying subcriteria and their hierarchization through the analytical hierarchy process (AHP), the technique for order of preference by similarity to ideal solution (TOPSIS), and a case study validation. Seven subcriteria were identified. The hierarchy of criteria was social (49.7%), technical (26.4%), and economic (23.9%). The tool involved: (i) users’ consultation about the perceived ease of use and availability of water sources other than rainwater; (ii) system dimensioning to establish supply size, maintenance requirements, and required water quality; and (iii) costs and benefits estimation. Tool validation in a rural area included the evaluation of the alternatives proposed: (a) alternative 1: potable domestic uses (PD) and non-potable (NPD); (b) alternative 2: PD and NPD, irrigation of crops and chicken farming for self-consumption; and alternative 3: PD and NPD and chicken farming for profit sale. The sensitivity analysis showed the tool’s consistency and robustness. Tool validation highlights the importance of integrating the three dimensions in selecting RWHS. The study provides a systematic methodology to assess and prioritize RWHS, appealing to policymakers, engineers, and practitioners facilitating water management and supply processes in rural areas.

**Supplementary Information:**

The online version contains supplementary material available at 10.1007/s11356-024-33734-8.

## Introduction

The world is facing increasing water scarcity that threatens people’s water provision (Islam et al. 2010; Imteaz et al. 2012) due to population growth (Nnaji et al. 2017; Lani et al. 2018), urbanization (Lani et al. 2018), and alteration of the hydrological cycles associated to climate change (Lani et al. 2018). These problems are especially critical for vulnerable regions in developing countries, such as rural areas, where water scarcity restrains people’s livelihoods (UNESCO 2020).

Water provision challenges have focused on decentralized water supply alternatives, including rainwater harvesting (RWH) (Muklada et al. 2016). In developing countries, RWH represents a reliable water source that is versatile and financially attractive compared to centralized systems. RWH could be the primary or complementary water source in water-scarce contexts to increase water availability (Aghaloo and Chiu 2020; Chiu et al. 2020; Khan 2023).

A key aspect of implementing RWH is related to people’s perception and acceptance since the operation and maintenance of these systems are highly influenced by users’ attitudes (Mankad and Tapsuwan 2011; Domènech and Saurí 2011; Domènech et al. 2013). The lack of technical knowledge (e.g., tank sizing, treatment design), operation and maintenance requirements (e.g., type and frequency of activities), and high installation costs of RWH systems (RWHS) have been identified as the main perceived barriers in different studies (Ward et al. 2013; Fuentes-Galván et al. 2018; Sheikh 2020). At the same time, external financial support and savings on water bills have been recognized as essential incentives (Ward et al. 2013).

Regarding technical aspects, most studies in RWH have focused on storage tank sizing since this is the most expensive and critical system component (Khan et al. 2017; Semaan et al. 2020). An oversized tank could increase investment costs and reduce water quality, while an undersized tank would fail to supply the water demand (Semaan et al. 2020). The design of RWHS in rural areas is challenging since water demand includes domestic and small-scale productive activities such as cultivation and livestock keeping (Domínguez Rivera et al. 2016; Fuentes-Galván et al. 2018; Saad and Gamatié 2020; Hoss et al. 2022).

Concerning economic aspects, the most popular indicators to assess the feasibility of RWHS are payback period, cost–benefit ratio, and net present value. The economic analysis includes costs such as investment, operation, and maintenance, avoided costs of water use, and environmental costs (Semaan et al. 2020). Typically, the analyses focus on the investment costs, while the environmental costs are rarely included due to the difficulties in their estimation (Rashid et al. 2016). Some researchers have concluded that a RWHS installation is not financially feasible if solely the investment and water savings costs are included in the analysis (Ghisi and Mengotti de Oliveira 2007; Chiu et al. 2009; Islam et al. 2010; Domènech and Saurí 2011; Roebuck et al. 2011; Christian Amos et al. 2016). Other researchers argue that RWH is financially feasible when external financial support is provided for implementation (Ghisi and Mengotti de Oliveira 2007; Islam et al. 2010; Mankad and Tapsuwan 2011; Santos and Taveira-Pinto 2013). Likewise, financial analyses have been carried out comparing an RWHS of a single-family household and a public building (Santos and Taveira-Pinto 2013), an RWHS of a single-family household with a multi-family building (Domènech and Saurí 2011), and an RWHS of commercial buildings with different sizes (Lani et al. 2018). The results of these studies show that typically, as building size increases, greater economic benefits are achieved.

The research on RWHS commonly addresses social, technical, or economic criteria independently. To the authors’ knowledge, no research has addressed integrally the three criteria to select a RWHS for a rural context. This comprehensive approach demands identifying the criteria weights to select a RWHS in a rural area, considering that people develop small-scale productive activities that increase water demand in these contexts. Multicriteria analysis (MCA) is a tool that facilitates selection processes by considering different alternatives and using an integral approach (Aznar and Guijarro 2012).

The analytical hierarchy process (AHP) is one of the MCA methods most widely used in water management. This method performs a hierarchical analysis of criteria, sub-criteria, and alternatives through pairwise comparisons to obtain a preferred alternative. The comparisons require extensive expert assessment (Motiee et al. 2023). Another method, ELECTRE, uses agreement and disagreement thresholds to determine the relative performance of alternatives and thus classify them. However, threshold selection could influence the results, which requires careful computation (Sulistianto et al. 2018). PROMETHEE is another MCA method based on comparisons by allocating preference values and output flow values, which allows for a classification to be obtained. However, assigning preference values can include biases, and the output flow values can be complex to understand (Isa et al. 2021). Other methods, such as TOPSIS and VIKOR, are considered simple since they are based on selecting the alternative closest to the positive ideal and furthest from the negative ideal. VIKOR uses utility functions to identify the solution with maximum group utility and minimum individual regret (Kahraman and Otay 2022). The selection with TOPSIS involves calculating Euclidean distances. TOPSIS is considered easy to understand, apply, and interpret (Li et al. 2022).

Furthermore, different research advocates for integral approaches that combine AHP with TOPSIS, VIKOR, ELECTRE, or PROMETHE as an alternative to reduce the need for expert input and generate synergies from the individual advantages of each method and even overcome their disadvantages. For instance, Li et al. (2022) used AHP and TOPSIS to classify internet-based platforms in the infrastructure sector. AHP was used to weight selection criteria and TOPSIS to rank the platforms. According to the authors, this combination was an effective assessment method that delivered stable, accurate, and objective results and prevented dispersion and subjectivity.

A search of recent literature (2017 February 2023) was carried out in Scopus and Science Direct, with the equation: TITLE-ABS-KEY ((“Rooftop rainwater harvesting” OR “Rainwater harvesting” OR “rainwater harvesting system”) AND (“multicriteria evaluation” OR “Multicriteria decision aid” OR “Multi‐criteria analysis” OR “Multicriteria decision analysis” OR “multicriteria” OR “Multi‐criteria”)). This search returned 71 relevant works. Seventy percent of these works (49) address identifying sites to install RWHS. Regarding MCA to compare hydraulic structures, researchers such as Loc et al. (2017) compared alternatives for integrated urban water management. Peters et al. (2019) selected the best potable water sources, and Reyes et al. (2018) compared alternatives to water mitigation. Despite the extended application of MCA in water management, only two studies were found that used this method to compare RWHS options: Hämmerling et al. (2020) compared different RWHS for urban areas in Poland suburbs, and Melville-Shreeve et al. (2016) selected RWHS in an urban area in a developed country. Therefore, this review shows the limited research regarding MCA for selecting RHW alternatives. No research using MCA to aid in selecting RWHS in rural areas from developing countries was found. In this context, our research question was centered around how to integrate social, technical, and economic criteria in a tool for selecting rainwater harvesting systems in rural areas. Our study aims to formulate a novel, validated tool to select RWHS in rural areas using multicriteria analysis and integrating social, technical, and economic criteria.

Results from this research could guide practitioners and decision-makers in assessing and prioritizing RWH alternatives using MCA. The guidance specifies the input data, procedures, and outputs or results. This guidance comprises a structured and systematic methodology describing the process of designing and selecting RWHS, applicable to rural areas, including water use for multiple purposes (domestic and small-scale productive uses). Furthermore, our study provides evidence to professionals from government agencies and policymakers to support policies and programs aligned with achieving the Sustainable Development Goals (SDG), which allow for reducing the existent gap between urban and rural areas regarding water for domestic use (SDG 6), the promotion of sustainable rural livelihoods (SDG 1), and resilience to climate change (SDG 13).

## Material and methods

**Fig. 1:**
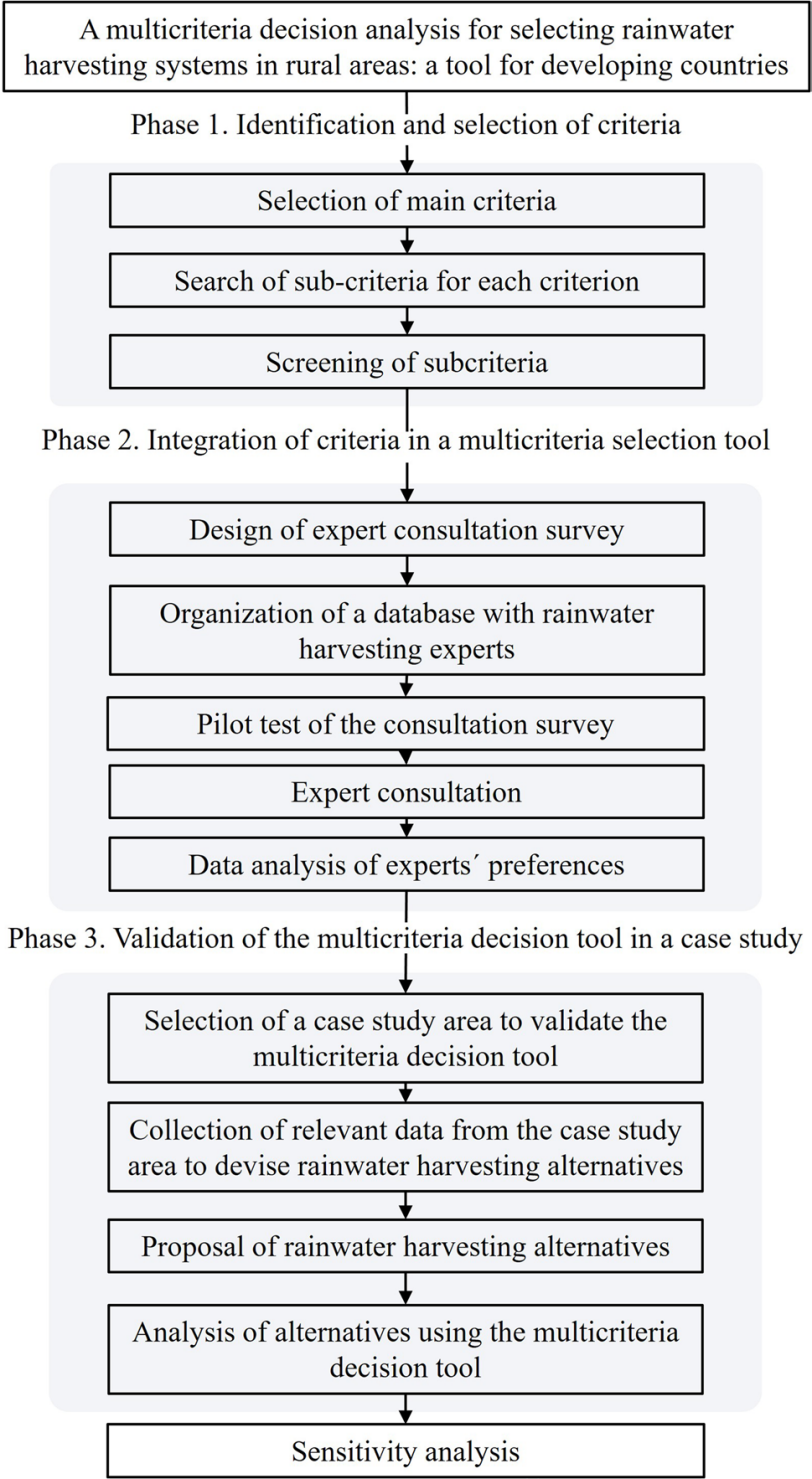
Methodology flowchart

This research was developed in three phases: (i) identification and selection of criteria, (ii) integration of criteria in a multicriteria tool, and (iii) tool validation in a case study (see Fig. 1).

### Development of the multicriteria selection tool

#### Identification of criteria and subcriteria

This study involved social, technical, and economic criteria that included subcriteria. Subcriteria and their measurement units were identified through a literature review in the academic databases Scopus® and Science Direct®. The search process comprised (i) search of economic and technical criteria since the economic analysis of a RWHS demands a technical design. The search keywords were “rainwater harvesting” and “rainwater harvesting system” combined with “economic analysis” and permutations of these terms (e.g., “investment feasibility analysis,” “economic feasibility”); and (ii) search of social subcriteria using the keywords “rainwater harvesting” and “rainwater harvesting system” combined with “receptivity” and permutations (e.g., “perception,” “acceptance”). The search was limited to scientific papers, including the terms in the title, abstract, or keywords. The resulting papers were filtered by reading (i) title, (ii) abstract, and (iii) content. A database was prepared in Excel® with the subcriteria found in the literature, the criterion, and a short description. A final list resulted from filtering subcriteria (see Table 3 in the Results section): (i) repeated, (ii) hard-to-quantify, (iii) mutually dependent, and (iv) calculated with the same variables.

#### Formulation of the multicriteria tool

The formulation of the multicriteria tool consisted of integrating the selected criteria and subcriteria through the multicriteria analysis approach. This approach supports decision-making by comparing alternatives to the criteria and subcriteria to select the most appropriate (Ammar et al. 2016). This study used AHP (Saaty 1980). The first hierarchical level was the criteria, the second was the subcriteria, and the third was the alternatives to be assessed.

Later, 99 experts (people with scientific publications on RWH) were selected to compare criteria and subcriteria. Experts were contacted by electronic mail enquiring about their availability to answer a survey to develop the pairwise comparison of criteria and subcriteria using the fundamental scale with nine intensity levels proposed by Saaty (Saaty 1980). Experts were selected to answer the survey through a video call by Meet® or independently through a Google Form®. Twenty-three experts answered the survey. Pairwise comparison matrices were prepared with the answers of each expert, and the consistency ratio ($\text{CR}$) was checked. An expert answer was considered valid if the $\text{CR}$ was lower than 10% (according to the matrix order) (Saaty 1980). Consistent answers from the surveyed experts were used to obtain an aggregated pairwise comparison matrix. This matrix was solved by normalizing the vectors of each column of the matrix and calculating the mean of the rows of the resulting matrix. This process allows determining the weight vector for all the criteria and subcriteria (Saaty 1980) (see Appendix A).

### Tool validation in a case study

#### Case study selection

**Fig. 2:**
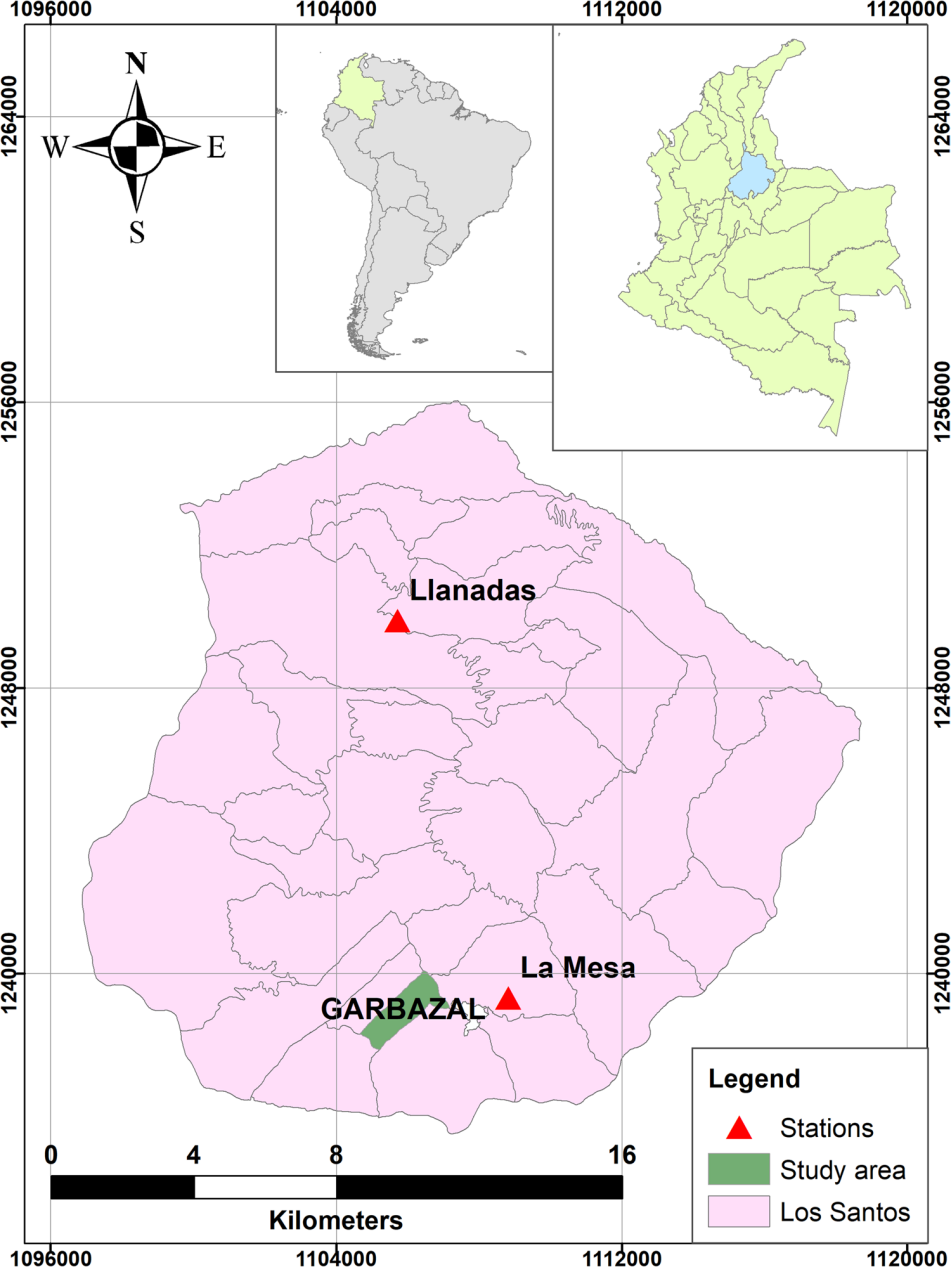
Location of the study area—Garbanzal

The multicriteria tool was validated in a Los Santos municipality (Santander – Colombia) rural settlement, Garbanzal village (see Fig. 2). Garbanzal had typical characteristics of rural areas, such as the use of multiple water sources, community-managed water systems, precarious coverage of water supply and sanitation services provided by local authorities, intermittent water services, and low water quality (Domínguez Rivera et al. 2016; Chino-Calli et al. 2016). Researchers such as Fuentes-Galván et al. (2018) and Hajani and Rahman (2014) report the extended use of RWH in rural areas, even as the primary water source where the access to water is deficient. This situation agrees with observations in Garbanzal, where RWH was a widespread alternative, typically as the primary water source. In addition, this village was selected due to factors such as access roads for vehicles and the willingness of its inhabitants to participate in the study. The village had an area of 1.97 km^2^, with 145 people living in 38 households (DANE 2016). Contact with the population was possible through a local water leader, a gatekeeper to link researchers with the community.

#### Collection of relevant information to validate the multicriteria tool

A workshop was conducted with key informants from the village to collect information on the study area regarding the number of households, their location, and water provision alternatives through social cartography following recommendations by Gilfus (2000).

A questionnaire was designed to collect data about RWH considering the social, technical, and economic aspects included in the multicriteria tool. The questionnaire had six sections: (i) demographic; (ii) household characteristics; (iii) available water sources, water use, and demand; (iv) perceptions about rainwater; (v) details of the existent RWHS (for people already using rainwater) such as technical characteristics, financial aspects, operation, and maintenance practices; and (vi) willingness to implement a RWHS and perceived benefits (for people without RWHS). The questionnaire is included in Appendix B. A pilot test was performed in June 2021, and the questionnaire was carried out in July 2021. The questionnaire was applied to household members aged 18 or older through face-to-face interviews after providing informed consent following guidelines of the ethics committee from Universidad Industrial de Santander. Twenty-nine completed surveys were obtained.

A water quality monitoring campaign was carried out to identify water treatment alternatives according to the characteristics of the study area. Representative water quality data was collected by taking rainwater samples in households with RWHS and various materials in the tank and roof. In addition, sampling was in November 2021, with the first rainfalls at the end of the dry season, when lower water quality is expected due to the accumulation of contaminants in the catchment area (roof) to capture the critical water quality values and propose a robust treatment for these conditions. Following recommendations by the World Health Organization (2012) and Gómez-Rozo and Silva-Lara (2019), point samples were collected once in 14 rainwater storage tanks of 14 households. Laboratory analyses, methods, and techniques used are summarized in Table 1.

**Table 1:** Laboratory methods and techniques for rainwater quality analyses in Garbanzal

Parameter	Method and technique
pH	SM 4500 H + B, Ed. 23rd
Turbidity	SM 2130 B (Ed. 23rd), nephelometric
Total iron	SM 3030 F, 3111 B (Ed. 23rd)—nitric and hydrochloric acid digestion. Spectrophotometry A.A. Direct air-acetylene flame
Electrical conductivity	SM 2510 B (Ed. 23rd)—electrometric
Nitrates	Methode par spectrometrie dabsortion moleculaire, 9e edition, J. Rodier 2009 L’Analyse de l’eau (French)
Total zinc	SM 3030 F, 3111 B (Ed. 23rd)—nitric and hydrochloric acid digestion. Spectrophotometry A.A. Direct air-acetylene flame
Residual chlorine	SM 4500 Cl G
Apparent color	SM 2120 C
Total coliforms	SM 9222 J (Ed. 23rd)—FPM
E. coli	SM 9222 J (Ed. 23rd)—FPM Membrane filtration

The hydroclimatic data was used to design the RWH alternatives. Rainfall analysis was used to estimate the tank size. Data on temperature, humidity, wind speed, and solar radiation were used to estimate crop irrigation demands. Finally, the design of gutters and downpipes was carried out using intensity–duration–frequency (IDF) curves. Data from these stations were checked to verify missing values. Table 2 summarizes the characteristics of the three hydroclimatic stations.

**Table 2:** Characteristics of the hydroclimatic stations used for dimensioning the components on the rainwater harvesting system alternatives in Garbanzal

Hydroclimatic station	Type	Temporal resolution	Data time interval	Distance to the study area	Responsible	Coordinates (N, E)
La mesa	Pluviometric	Day	1974 – 2021	2 km	IDEAM	1239314, 1108828
Llanadas	Meteorologic	30 min	2020 – 2022	10 km	GPH^a^	1249877, 1105726
El Cucharo	Pluviometric	Day	1965 – 2009	27 km	IDEAM	12499897, 1105635

*IDEAM*, Instituto de Estudios Ambientales y Meteorológicos de Colombia; ^a^*GPH*, Research group on water resources and sanitation from Universidad Industrial de Santander

#### Formulation of rainwater harvesting system alternatives for Garbanzal

Three RWHS alternatives were proposed according to the specific characteristics of the study area (water demand for domestic uses, crop irrigation, and livestock farming) obtained from the data collection strategies implemented, including household survey, hydroclimatic data analysis, water quality sampling: (i) alternative 1: domestic needs for potable use (PD) and non-potable use (NPD); (ii) alternative 2: PD and NPD, irrigation of crops for self-consumption (ICS), and chicken farming for self-consumption (ChS); and (iii) alternative 3: PD, NPD, and chicken farming for both self-consumption (ChS) and for profit sale (ChM). Alternatives 2 and 3 comprised the same components.

Water demand was estimated considering the typical domestic and small-scale productive activities in the study area. The water demand for crop irrigation was estimated using the CROPWAT software (https://www.fao.org/land-water/databases-and-software/cropwat/es/). Water demand for other uses was estimated based on information from the literature (MinDesarrollo 2000; FAO 2000). The catchment area was the household roof, considering the size and materials typically found in Garbanzal and recommendations from the literature (Unatsabar 2001; Abdulla and Al-Shareef 2009). Gutters and downpipes were sized based on the open-channel flow theory and standards from the Código Colombiano de Instalaciones Hidráulicas y Sanitarias (ICONTEC 2017). The materials were chosen based on recommendations from the literature (Unatsabar 2001). The tank was sized using the mass balance method THETA (Fewkes and Butler 2000), which provides an intermediate size between the yield after spillage (YAS) method (Fewkes and Butler 2000) and yield before spillage (YBS) method (Fewkes and Butler 2000) (see Appendix C).

The distribution network was designed using the modified Hunter method and the layout for a household with the typical characteristics of the study area. The maximum probable flow was calculated, and the pipes of the distribution network were dimensioned to fulfill velocity requirements in each section and allowable end pressure in the water devices. This information was used to calculate the input pressure and required pump characteristics.

Regarding the treatment of rainwater, three barriers were proposed based on the results of the laboratory tests summarized in Appendix G. First, prefiltration consisted of a mesh at the entrance of gutters and downpipes and a first-flow diverter. Second, filtration comprises a homemade slow sand filter inside the household kitchen to provide water for the uses that demand potable water. This treatment system was defined considering the results of the water quality analysis that determined that the raw water in the area did not demand preliminary treatment (e.g., coagulation, flocculation, and sedimentation) (Ramírez and Pérez 2002). The proposed treatment did not require pumping, and it is a physical and biological treatment commonly used in rural areas due to its ease of use and construction (López 2020). The third barrier was disinfection through boiling for domestic uses that demand potable water.

#### Selection of the rainwater harvesting system for Garbanzal using the multicriteria tool

Once the three RWHS alternatives were designed and the weight vectors obtained through AHP (Saaty 1980), TOPSIS was used to establish the most appropriate alternative for the case study, following methods described by Soto-Paz et al. (2019). For this, the behavior of each alternative was established for each selected subcriteria, building a normalized decision matrix and multiplying the corresponding weight. Then, the ideal positive and negative solutions were established, and the Euclidian distance between the alternatives to these two solutions and the relative proximity of each alternative to the ideal solution were calculated. A ranking of the alternatives was obtained starting from the distances, placing the most favorable for the case study in the first place.

### Sensitivity analysis

A sensitivity analysis was carried out to establish the stability of the model results when the input parameters were modified (Barredo-Cano 1998), that is if the same alternative was selected when changing the criteria weights. The weight of the first criterion was changed from 1 to 100%, and, for each of these variations, all possible combinations of weights were assigned to the other two criteria, all with 1% intervals. Then, the same procedure was carried out for the second and third criteria, obtaining all the possible combinations of weights for the criteria. These combinations were generated using Python®, and with each combination, the TOPSIS method was applied, using Excel®, to obtain the ranking of alternatives for each modification.

## Results and discussion

### Development of the multicriteria selection tool

#### Identification of criteria and subcriteria

Fifty-one papers were selected and fully read, resulting in 294 assessment subcriteria (Appendix D). Table 3 shows the selected subcriteria, identifier (ID), the corresponding criteria, metric, and description.

**Table 3:** Summary of the assessment subcriteria for selecting rainwater harvesting systems in rural areas

Criteria	ID	Subcriteria	Metrics	Description
Social	S.1	Perceived ease of use	5 categories	Ease of use perceived by people. Categories: very hard (1), difficult (2), normal (3), easy (4), and very easy (5)
	S.2	Perceived availability of water sources other than rainwater	5 categories	Perception of the amount of water available from sources other than rainwater. Categories: very low (1), low (2), medium (3), high (4), and very high (5)
Economic	E.1	Benefit	COP	Benefits of the RWHS throughout its lifespan
	E.2	Cost	COP	Costs of the RWHS throughout its lifespan
Technical	T.1	Supply size	m^3^	Maximum volume of rainwater that can be collected in a specified area during a specified period
	T.2	Comparison of the water quality provided for the rainwater harvesting system and other alternatives of supply	Score (0 – 5)	People’s perceptions when comparing the quality of water offered by available water sources other than rainwater and the quality of water delivered by the rainwater harvesting system are as follows: Rating from 0 to 5, assigned according to the rules described in “Selection of alternatives using the multicriteria tool”section
	T.3	Maintenance	5 levels	Maintenance requirements of the rainwater harvesting system throughout its lifespan. Difficulty levels: very low (1), low (2), medium (3), high (4), and very high (5)

In the social criterion, two subcriteria were identified: perceived ease of use and perceived availability of water sources other than rainwater (Emenike et al. 2017; Ignacio et al. 2019). Subcriterion S1 is the direct people’s perception of the ease of use of the proposed RWHS alternatives. This perception is relevant since this could positively or negatively affect the use intention behavior of system users, which influences its design, operation, and maintenance due to the close user-system interaction (Ignacio et al. 2019). Criterion S2, which measures the perception of the availability of other water sources different from the RWHS, was based on the people’s perception of water quantity offered by other water sources available and that offered by the RWHS. This subcriterion could directly influence the design and implementation of a RWHS since a low perception of water availability from other sources could ensure a generalized use of rainwater. In contrast, perceiving high water availability from other sources could eliminate the need for a RWHS (Emenike et al. 2017).

In the economic criterion, the cost/benefit ratio was proposed since this is one of the most widely used indicators for the economic feasibility of RWHS (Semaan et al. 2020). However, it was decided to use two separate criteria, E.1 Benefits and E.2 Costs, to independently make explicit the amount of costs and benefits of the RWHS.

For the technical criterion, the subcriterion T.1 supply size is the maximum rainwater volume that can be collected in a roof area in a specific period. This metric was proposed by Abu-Zreig et al. (2019) and helps involve two critical parameters required to calculate tank size, which are considered by several researchers (Chiu et al. 2009, 2015; Campisano and Modica 2012; Bocanegra-Martínez et al. 2014; Allen and Haarhoff 2015; Khan et al. 2017; Fonseca et al. 2017; Lopes et al. 2017): rainfall and roof area. Subcriterion T.2, a comparison of the water quality provided for the rainwater harvesting system and other supply alternatives, was proposed to assess the system’s water quality using some of the parameters suggested by Abdulla and Al-Shareef (2009). However, measuring this quality would require systems in operation that are incompatible with a tool to select the most appropriate alternative to be implemented in a specific context. Due to this, it was decided to modify the metric of this sub-criterion without losing the purpose of incorporating aspects of water quality. Therefore, the rating of this sub-criterion was based on people’s perception when comparing the quality of water offered by available water sources other than rainwater and the quality of water delivered by the RWHS. For instance, a perception of poor water quality in the available sources other than rainwater would result in a higher rating in the RWHS alternatives that can offer water for uses that require better quality. In comparison, a perception of good quality in sources other than rainwater would have a lower rating in the RWHS alternatives that can offer water for uses requiring better quality.

Subcriterion T.3, maintenance, is proposed by Melville-Shreeve et al. (2016), even though they did not specify the metrics for the assessment. In this case, a Likert scale of five points was proposed, breaking down the maintenance difficulty into four items: (i) ease of obtaining the necessary tools and equipment; (ii) qualification of the personnel required to carry out the activities; (iii) required annual frequency of activities; and, (iv) ease of obtaining the necessary materials. A database of possible components of a RWHS was prepared. Each component’s maintenance difficulty level was calculated as the average of the difficulty level of the four items mentioned above. Finally, to assign a score for this sub-criterion for each alternative, the components of the alternative were searched in the database, and the score of the component with the highest difficulty level was assigned. The rating for each of the four items for components and alternatives appears in Appendix E.

#### Formulation of the multicriteria tool

The criteria and subcriteria were weighed based on the preference of 15 experts from Mexico, Colombia, Argentina, Costa Rica, and Spain, of which 80% had technical, and 20% had economic expertise. This number of consistent answers is adequate as those were obtained from people with competencies in RWH. Other studies using multicriteria analysis tools, for instance, Saeedi et al. (2022), Galarza-Molina et al. (2015), and Melville-Shreeve et al. (2014) relied on the participation of between nine and 15 experts to compare criteria and subcriteria. Table 4 includes the aggregated pairwise comparison matrix for criteria and subcriteria.

**Table 4:** Aggregated pairwise comparison matrix for criteria and subcriteria

	Social		Economic		Technical
Social	1		2		2
Economic	1/2		1		8/9
Technical	1/2		9/8		1
			Perceived ease of use	Perceived availability of water sources other than rainwater	
Perceived ease of use			1	3/2	
Perceived availability of water sources other than rainwater			2/3	1	
		Benefits		Costs	
Benefits		1		7/2	
Costs		2/7		1	
	Supply size		Comparison of the water quality provided for the rainwater harvesting system and other alternatives of supply		Maintenance
Supply size	1		4/3		9/5
Comparison of the water quality provided for the rainwater harvesting system and other alternatives of supply	3/4		1		9/7
Maintenance	5/9		7/9		1

The weighting of criteria and subcriteria shown in Table 5 evidences that although 80% of the answers were from experts with technical backgrounds, there was a tendency to allocate more importance to the social criteria. These results can be attributed to the acknowledgment by the experts that the character of a decentralized system with the use, operation, and maintenance being managed by the owners is highly influenced by the users’ attitudes (Mankad and Tapsuwan 2011; Domènech and Saurí 2011; Domènech et al. 2013). On the other hand, the perceived ease of use had a higher weight than the perception of availability of water sources other than rainwater. A possible explanation of this marked difference could be that experts recognize that even if rainwater is required to ensure water availability, if the RWHS is considered challenging to operate by users, this would prevent a generalized adoption, as suggested by Ignacio et al. (2019).

**Table 5:** Weights of economic, social, and technical criteria and subcriteria

Criteria	Weight	ID	Subcriteria	Local weight	Global weight
Social	49.7%	S.1	Perceived ease of use	61.1%	30.4%
		S.2	Perceived availability of water sources other than rainwater	38.9%	19.3%
Economic	23.9%	E.1	Benefits	77.8%	18.6%
		E.2	Costs	22.2%	5.3%
Technical	26.4%	T.1	Supply size	43.4%	11.4%
		T.2	Comparison of the water quality provided for the rainwater harvesting system and other alternatives of supply	32.0%	8.4%
		T.3	Maintenance	24.6%	6.5%

Regarding economic subcriteria, the benefit had a higher weight than the cost. Even though experts knew that the economic subcriteria were assessed exclusively in monetary terms, the preference for the benefits could be regarded as the experts’ recognition of the multiple benefits of RWHS that frequently are hard to quantify in monetary terms, as has been previously mentioned (Chiu et al. 2009; Christian Amos et al. 2016).

Concerning the technical criterion, supply size was weighted as the most crucial subcriterion, consistent with previous studies (Khan et al. 2017; Semaan et al. 2020). Experts can appreciate the importance of this subcriterion since it is highly related to the amount of water provided by each alternative and directly affects the system’s efficiency and feasibility.

### Tool validation in a case study

#### Collection of relevant information to validate the multicriteria tool in Garbanzal

- Population characteristics
  Twenty-nine households from the existing 38 answered the survey. The household size was four people. The available water sources were rainwater, a centralized water system from a surface source, a centralized water system from a groundwater source, and a water tanker truck. People indicated none of these sources, except rainwater, was available all year round.
  Regarding the existent rainwater harvesting systems, some households collected water from more than one roof, and these roofs could be made from different materials (sheet metal—29%, fiber cement—29%, and ceramic tile—26%). The gutters and downpipes were not massively adopted (14% and 69%, respectively). Only 13% of households had meshes and filters, mainly at the storage tank inlet. The households had between one and four storage tanks, with volumes between 500 and 2000 L. Water was used for domestic uses and small-scale productive activities for self-consumption and sale (cultivation of maize, cassava, avocado, and plantain, and keeping chickens, cattle, horses, sheep, goats). The features of access to water in the study area were consistent with the typical characteristics of rural contexts (Domínguez Rivera et al. 2016; Chino-Calli et al. 2016), such as community-managed multiple water sources in response to the precarious coverage of water and sanitation services provided by local governments, service intermittence, and poor water quality. This situation shows the relevance of RWHS in the studied context as reported in other rural areas where, given the scarcity or precariousness of centralized systems, people relied on rainwater as their primary water source (Fuentes-Galván et al. 2018).
- Hydroclimatic data
  The study area experienced an annual decreasing trend in rainfall in the last 40 years, from 1008 mm (1974–1979) to 740 mm in the last decade (2010–2019), a total reduction of 270 mm (see Fig. 3). The decrease in the amount of rainfall in the area is likely an effect of climate change (Campisano and Modica 2012) since these results are from the analysis of a data series of 45 years.
  The study area has a bimodal rainfall pattern with two rainy seasons (April–May and September–October) and two dry seasons (June–July and December–January), associated with the Intertropical Convergence Zone (see the annual cycle of rainfall in the study area in Appendix F). The rainfall variability could affect the availability and distribution of water. Regarding availability, the progressive rain reduction could represent a decrease in rainwater storage needs. On the other hand, seasonality affects distribution since, throughout the year, there will inevitably be spills and periods when the collected water is insufficient to supply the demand. These factors affect the RWHS reliability since the system will depend on a water source that is not constant throughout the year. Therefore, the RWHS design considered these factors and looked for a balance between maximum possible reliability and reasonable tank size.
- Rainwater quality in Garbanzal
  **Fig. 3:**
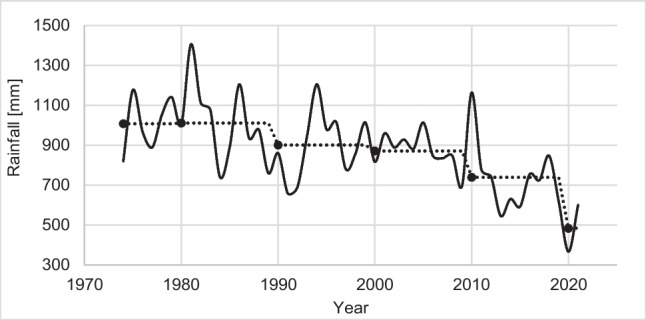
Average annual rainfall series for each decade at the “La Mesa” station (1974 – 2021). Note: The blue line is the annual accumulated rainfall, and the red line is the average rainfall for each decade
  All the physicochemical parameters fulfilled the WHO water quality standards for human consumption (OMS 2011) (See Appendix G). However, turbidity (1.94 ± 1.31 NTU) and electrical conductivity (70.40 ± 36.84 μs/cm) values showed the need to implement a treatment to improve the water quality for human consumption. The iron (0.054 ± 0.01 mg/L) and zinc values (0.052 ± 0.06 mg/L), although low, may be associated with the leaching of materials from metal roofs or with the combustion of coal or other fossil fuels for cooking (Gómez-Rozo and Silva-Lara 2019). In comparison, the nitrate values (0.932 ± 0.98 mg/L) show contamination possibly associated with factors such as the combustion of biomass or fossil fuels or wet or dry deposition of animals on roofs (Gómez-Rozo and Silva-Lara 2019). The residual chlorine values (non-detectable) show no disinfection practices in the storage tanks. Regarding microbiological parameters, total coliforms (1046 ± 1933 CFU/100 ml) and *E. coli* (33 ± 45 CFU/100 ml) did not meet WHO standards, and their presence could be linked to bird droppings deposited on household roofs (Gómez-Rozo and Silva-Lara 2019). Some recommendations to improve rainwater quality include covering the storage tank, frequently performing cleaning and maintenance activities, and installing the primary treatment.

#### Formulation of rainwater harvesting systems alternatives for Garbanzal

**Fig. 4:**
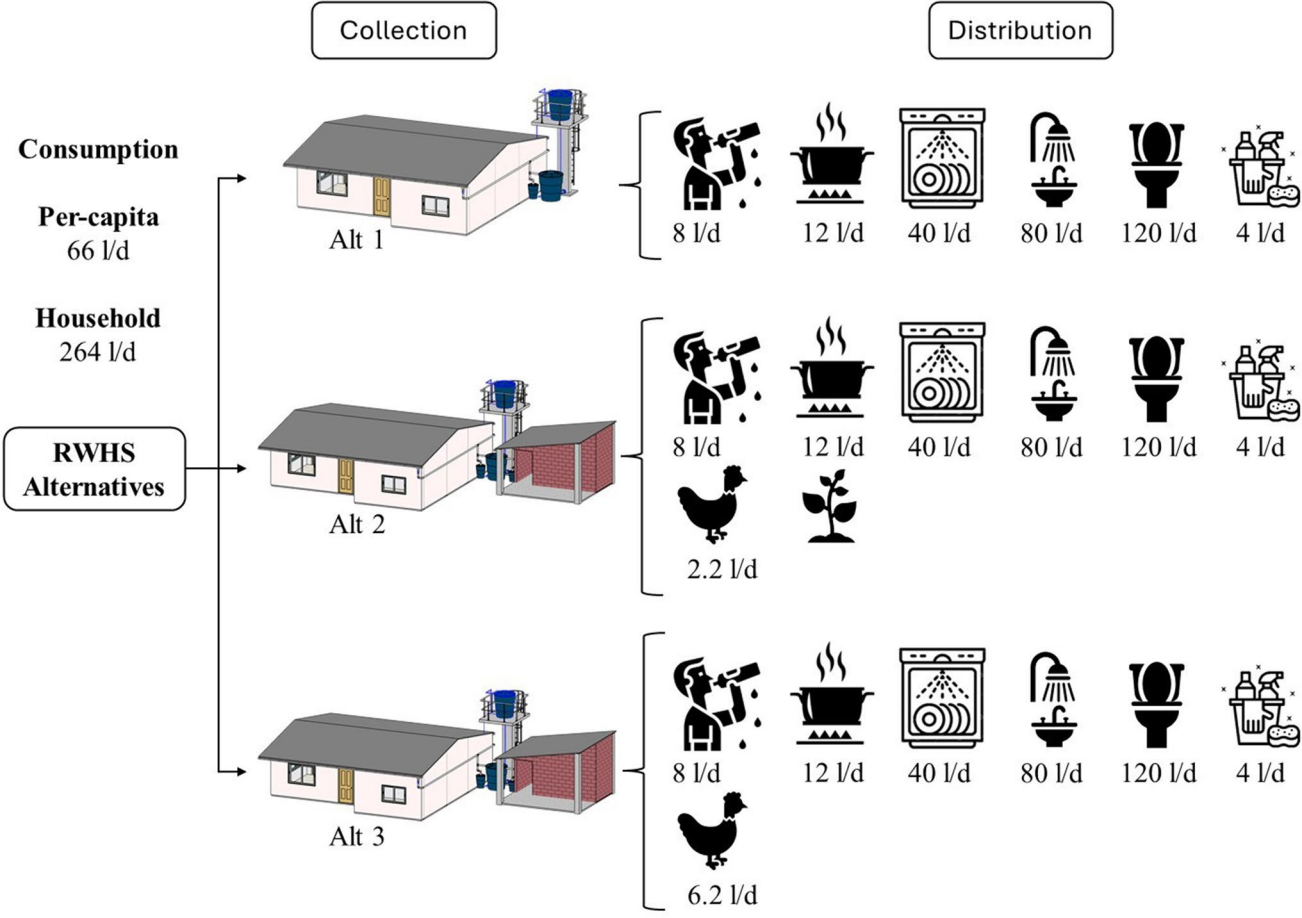
Water uses and demand on each proposed RWH alternative for Garbanzal. Note: The water demand for crops was estimated using the CROPWAT software. For this reason, there is no single demand value but an irrigation schedule. Thus, irrigation demand is not specified in the figure

The design of the three alternatives was based on the study area’s characteristics and literature recommendations. Design characteristics of the three proposed alternatives for the Garbanzal context are shown in Fig. 4 and Table 6. Water for domestic activities included drinking, preparing food, washing dishes, showering and hand washing, toilet flushing, and cleaning. Livelihood activities included irrigation of a maize crop and keeping chickens for self-consumption and sale.

**Table 6:** Design characteristics of the proposed alternatives of rainwater harvesting systems for Garbanzal

	Alternative 1	Alternative 2	Alternative 3
Uses	PD^a^, NPD^b^	PD^a^, NPD^b^, ICS^c^, ChS^d^	PD^a^, NPD^b^, ChM^e^, ChS^d^
Demand PD	15 lpcd^f^	15 lpcd^f^	15 lpcd^f^
Demand NPD	56 lpcd^f^	56 lpcd^f^	56 lpcd^f^
Demand ICS	-	Results of the CROPWAT software for a maize crop of 105 m^2^. Two cycles per year	-
Demand ChS	-	11 chickens – 0.2 l/chicken*day	11 chickens – 0.2 l/chicken*day
Demand ChM	-	-	20 chickens – 0.2 l/chicken*day
Roof area	100 m^2^	125 m^2^	125 m^2^
Gutters	Height: 12 cm. Width: 6 cm	Height: 12 cm. Width: 6 cm	Height: 12 cm. Width: 6 cm
Downpipes	Household roof: 3” Warehouse roof: 0	Household roof: 3” Warehouse roof: 2”	Household roof: 3” Warehouse roof: 2”
Storage	1 elevated tank: 1000 L 1 overground tank: 1000 L	1 elevated tank: 1000 L 1 overground tank: 1000 L	1 elevated tank: 1000 L 1 overground tank: 1000 L
Primary treatment	Mesh in gutters Mesh in downpipes First-flush diverter	Mesh in gutters Mesh in downpipes First-flush diverter	Mesh in gutters Mesh in downpipes First-flush diverter
Secondary treatment	Slow sand filter	Slow sand filter	Slow sand filter
Tertiary treatment	Boling	Boling	Boling

^a^PD, Domestic uses that require potable water; ^b^NPD, Domestic uses that require non-potable water; ^c^ICS, water demand for irrigation of crops for self-consumption; ^d^ChS, demand of chickens for self-consumption; ^e^ChM, demand of chickens for sale. ^f^lpcd, liters per capita per day

Table 6 shows the proposed uses for each alternative and corresponding water demands for potable and non-potable use, irrigation, and chicken farming. The dimensions of the RWHS components are specified: roof area, gutter and downpipes, tank, and treatment system. The tank sizing analysis, including efficiencies, can be found in Appendix H, and the detailed description of all the RWHS components and their dimensions can be found in Appendix I.

#### Selection of alternatives using the multicriteria tool

Table 7 shows the score of each alternative for the selected subcriteria and criteria. Regarding the social subcriteria, the perceived ease of use score for the three alternatives was between average and easy. This behavior can be associated with the similarity of the technological components of the three alternatives. Concerning the perceived availability of water sources other than rainwater, people assigned a low score, for which the RWHS alternative that offered more water had a higher score. Thus, alternative 1 had the lower score since it provided less water than alternatives 2 and 3 (i.e., 2 and 3 provide the same amount of water).

**Table 7:** Selection matrix for rainwater harvesting system alternatives for Garbanzal

	E.1. Benefit^a^ [USD]	E.2. Cost^a^ [USD]	S.1. Perceived ease of use [Likert scale]	S.2. Perceived availability of water sources other than rainwater [Likert scale]	T.1. Supply size [m^3^]	T.2. Comparison of the water quality provided for the rainwater harvesting system and other alternatives of supply Score [0–5]	T.3. Maintenance [Likert scale]	Score
Alt. 1	$ 1545	$ 1841	3.24	1	57.87	4	3	0.320
Alt. 2	$ 16,930	$ 12,641	3.10	2	72.34	4	3	0.324
Alt. 3	$ 49,742	$ 34,162	3.10	2	72.34	4	3	0.681

*Alt*., alternative; ^a^representative market rate: 1 US dollar = 4180.99 Colombian pesos

Concerning the economic subcriteria, the costs involved in the analysis were investment, operation, maintenance, and production costs of the small-scale agricultural activities. For chicken farming, it was assumed that people kept chickens permanently throughout the year, in semi-annual cycles, where young animals were acquired initially and produced eggs throughout the cycle. The chickens were sold at the end of the cycle. For agricultural production, a crop calendar was established with two cycles per year, adjusted according to crop stage, considering that the highest water requirement was synchronized with the periods of greatest rainfall in the year, aligned with the cultivation practices in the study area. The benefits included were the avoided costs of water from the centralized system for these activities and the income obtained from crops and animals. Appendix J present the costs and benefits cash flow.

Finally, concerning the technical subcriteria, the supply size was assessed for the catchment area of each alternative during a year. For this, the year with higher water offer was used since this subcriterion refers to the maximum volume of water that a specific area can collect during a specific period. This value is the volume collected if all rainfall could be captured without spills, assuming “infinite” storage (Abu-Zreig et al. 2019).

The score from comparing the water quality of the RWHS and other water sources was determined through a comparative analysis of people’s perceptions about the water quality offered by available water sources different from rainwater (O) and the water quality offered by the RWHS. Scores from 1 to 5 were assigned from O and RWHS, 1 for very low water quality, and 5 for very high water quality. The scoring followed these rules: if water quality from the RWHS was perceived lower than O, the assigned score was 0; if water quality from the RWHS was perceived equivalent to O, the assigned score was 1; and if water quality from RWHS was perceived better than O, the assigned score was the result of a mathematical operation ((RWHS-O) + 1). In the case study, the perceived water quality offered by all water sources besides rainwater was low. In contrast, the perceived water quality from the three proposed RWHS alternatives was very high. Thus, the score of this subcriterion was equal for the three assessed RWHS alternatives, 4. The maintenance subcriterion was assessed by scoring the maintenance difficulty of all system components and assigning the component’s value with the higher difficulty value to the alternative. In this case study, the pump was the component with the highest difficulty in maintenance. However, the three proposed alternatives included a pump and, thus, obtained the same score in this subcriterion.

The weights of criteria and subcriteria and the decision matrix were used as inputs to rank the alternatives. Thus, the best alternative for the study area was alternative 3 (see Table 7).

### Sensitivity analysis of results

It was interesting to verify if alternative 3 continued to be the most appropriate with changes in the weighting of the criteria and under what conditions this was reversed. For this, 5151 iterations were performed with all possible criteria weightings. It was found that, in 85% of the cases, alternative 3 had the highest score. In 14%, alternative 1 had the highest score, while only in 1%, alternative 2 had the highest score. The scenarios where alternative 1 was preferred were associated with a greater weighting of the social criterion. In none of the iterations, alternative 2 obtained the highest score. The sensitivity analysis results indicate the proposed tool is stable when the criteria weights vary. Appendix K show the score charts for each alternative by varying the weights of each criterion from 0 to 100%.

### Discussion of the applicability of the multicriteria selection tool of rainwater harvesting systems for rural areas

The proposed multicriteria tool provides a structured procedure to assess and select RWHS considering technical, social, and economic criteria. This process facilitates decision-making for complex situations, such as when planning the implementation of RWH. The multicriteria selection tool of RWHS for rural areas provides a solution to address global challenges related to water provision, particularly for the rural world, where water uses are diverse, multiple water sources are used, the required water quantity could be higher compared to urban areas, and water allocated for small-scale productive uses is crucial for people’s well-being. Since RWHS are relevant to water alternatives in these regions, it is essential to rely on selection processes that integrate social, technical, and economic criteria to ensure sustainable decisions. The tool proposed in this study identified criteria and subcriteria used in the literature. It developed an objective and systematic weighting process, using AHP, that considered the opinions of various experts in the field. This selection approach allows decision-makers to assess multiple criteria and alternatives in a structured and objective manner.

The proposed tool has a wide applicability range. It could be used by planners, engineers, and other professionals to design and implement integrated water resource management projects to assess and prioritize RWHS alternatives in rural contexts. The tool has been formulated to facilitate decision-making considering principles of efficiency and sustainability, adapted to contexts needs and particularities, and provide a sound and structured methodology to address the design of RWHS. It also facilitates decision-making in government institutions and public policy planners, a sound base for developing policies and strategies related to water management in rural areas.

The multicriteria tool fulfills five guiding principles related to the sustainability of water and sanitation technological systems stated by the Colombian regulation for rural areas (Ministerio de vivienda ciudad y territorio): (i) implementation of appropriate technological alternatives after assessing criteria such as perceived ease of use, benefits, and costs, ensuring solutions are simple, adequate, and cost-effective according to the community needs; (ii) operational sustainability by assessing maintenance difficulty, ensuring that owners can operate and maintain the system in the long-term; (iii) water availability is also considered since it assesses supply size and end-uses to ensure water quantity and quality requirements; (iv) environmental sustainability by assessing criteria related to the preservation of water and soil, inherent advantages of RWHS; and (v) community participation since the tool involved users’ opinions in the comparison of criteria and subcriteria, actively involving community in the final selection of the alternatives.

**Fig. 5:**
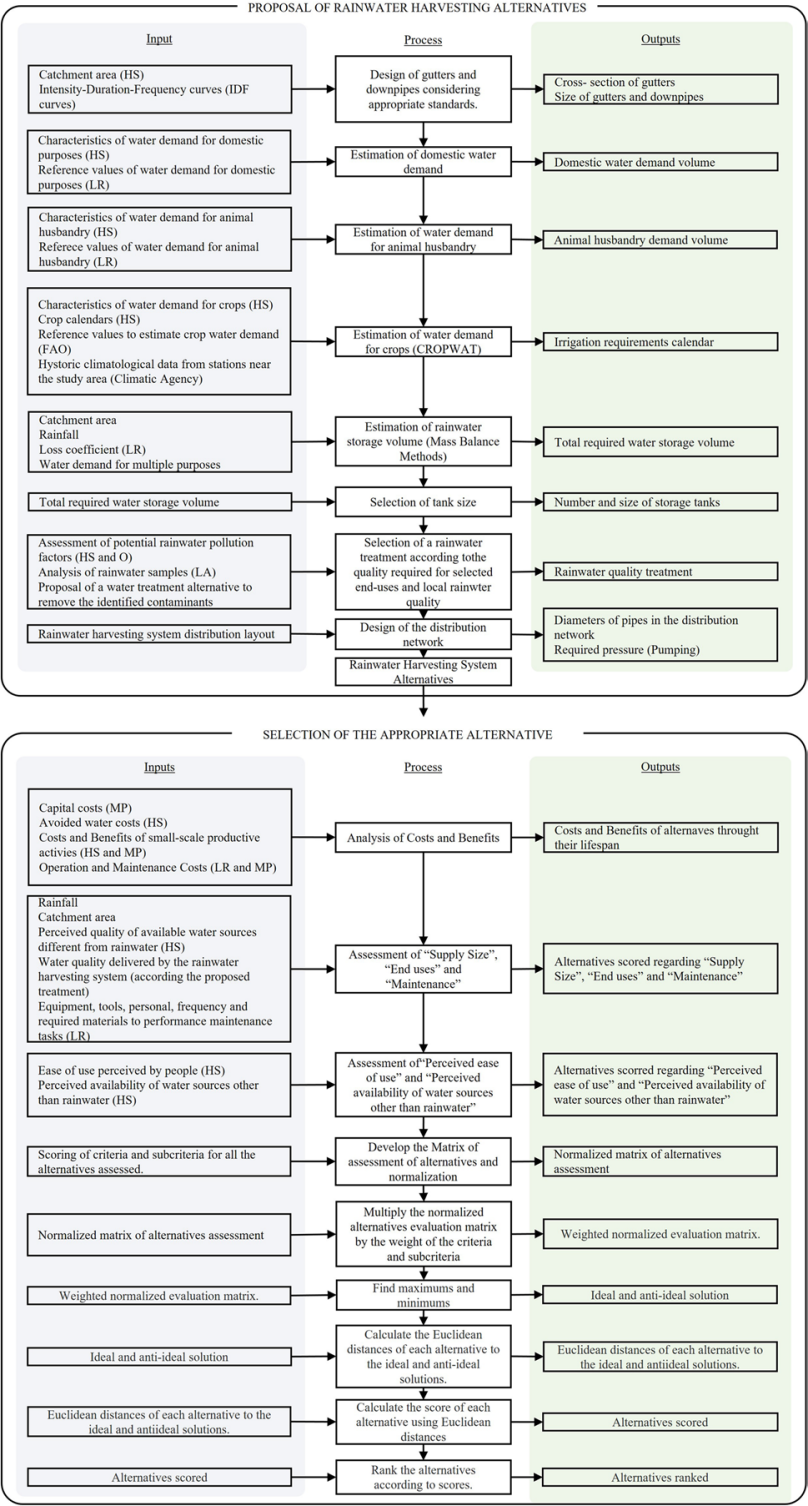
Flowchart for the proposal and selection of rainwater harvesting systems in rural areas using multicriteria analysis. Notes: HS, household survey; LR, literature review; O, observation; LA, laboratory analysis; MP, market prices

The tool also significantly contributes to the Sustainable Development Goals (SDGs), specifically ODS 6, “Clean water and sanitation,” by providing a practical methodology for selecting RWHS that improves water and sanitation, considering accessible costs and social criteria that favor community water management; ODS1, “no poverty” by providing water for small-scale productive activities that promote people’s livelihoods; ODS3 “good health and well-being” ensuring access to water which is an essential service for people to prevent water-borne diseases; ODS 11 “Sustainable communities and cities,” since it supports the adoption of RWHS to improve the efficient use of water, considering ease of use and maintenance difficulty and it ensures sustainability over time. Finally, it aligns with ODS 13 “climate action” by enhancing the implementation of solutions that encourage climate change adaptation by contributing to reducing floods and users’ dependency on traditional water sources. The tool can be upgraded by incorporating environmental criteria and subcriteria involving the environmental impacts of the RWHS alternatives that can influence the final selection of alternatives in a specific context. Figure 5 shows a flowchart summarizing the steps to apply the proposed tool in other rural contexts, considering each implementation site’s input data and specific characteristics. We understand our proposal as a tool since we provide a structured and systematic way of carrying out a task, in this case, the selection of a RWHS for a rural area. In addition to the process, we provide information to collect relevant information, recommendations, standards and design procedures, and criteria with weights based on expert opinion that facilitate decision-making.

## Conclusion

This study proposes a selection tool for rainwater harvesting alternatives for rural contexts, considering social, technical, and economic criteria and subcriteria resulting from an extensive literature review. The significance of these criteria was established using the analytical hierarchy process, which allows the identification of the most relevant criteria for decision-making: social (49.7%), technical (26.4%), and economic (23.9%).

The tool application combines inputs from users and experts. Users’ participation addressed the assessment of social subcriteria: perceived ease of use and availability of water sources other than rainwater. The experts assessed the social, technical, and economic criteria and the associated subcriteria. The integration of the contributions of experts and users defines the most appropriate alternative for a specific context.

Tool validation showed a practical and objective approach for the sustainable use of water resources since their results are compelling, providing the RWHS alternative that is more appropriate for a specific context and goals since it considers expert preferences with different opinions. Tool results are steady concerning changes in weights, which increases reliability.

The tool presented allows for designing, assessing, and prioritizing RWHS alternatives using social, technical, and economic criteria. Thus, it can be adapted to specific project needs. This tool could support the development of policies and strategies around water management in rural areas since it is aligned with the principles of Colombian water supply and sanitation regulations for these contexts and contributes to the achievement of different targets of the Sustainable Development Goals, particularly, 3, 6, 11, and 13. An improvement to the proposed tool would be incorporating environmental approaches for more sounding and integral results.

Our study has limitations. For instance, the study area used for validation was relatively homogeneous in its characteristics. This homogeneity influenced the proposal of similar alternatives, which may represent a limitation of this study. For this reason, it is suggested that the tool be validated in different contexts in future research. On the other hand, given the increasing recognition of involving environmental criteria as part of decision-making processes, it is suggested that environmental sub-criteria be explored to have a more holistic decision process. Finally, another limitation was the configuration of the experts’ panel, composed mainly of academics with technical expertise outside the validation context. Future research should involve a more diverse expert panel for a more integral tool. Overcoming these limitations would increase the usefulness and robustness of the tool and its application potential in various contexts.

### Supplementary Information

Below is the link to the electronic supplementary material.

Supplementary file1 (DOCX 881 KB)

## Data Availability

Data supporting reported results can be found in the appendices.
