# Supplementary material for: A multicriteria decision analysis for selecting rainwater harvesting systems in rural areas: a tool for developing countries
Source: Environ Sci Pollut Res Int. 2024 Jun 14;31(29):42476–91. doi: 10.1007/s11356-024-33734-8 (PMC11219541; doi:10.1007/s11356-024-33734-8)
Supplement: Supplementary file 1 — Supplementary file1 (DOCX 881 KB) [file 11356_2024_33734_MOESM1_ESM.docx]

**Multicriteria decision analysis for selecting rainwater harvesting systems in rural areas**

Prieto-Jiménez, D. ^a^; Oviedo-Ocaña, E.R. ^ab^*, Gómez-Isidro, S.^c^; Domínguez, I.C. ^ad^

^a^ Escuela de Ingeniería Civil

Facultad de Ingenierías Físico-mecánicas

Universidad Industrial de Santander

Carrera 27 Calle 9, Bucaramanga, Colombia

^b^ https://orcid.org/0000-0002-8970-7322

^c^ https://orcid.org/0000-0002-9200-6586

^d^ https://orcid.org/0000-0002-7677-2731

*Corresponding author: Oviedo-Ocaña, E.R. Universidad Industrial de Santander, eroviedo@uis.edu.co

Word count: 10,086

**Appendix A.** **Calculation of the Consistency Ratio and Weight Vector**

Calculation of the consistency ratio ($CR$) for the experts' responses, aggregated pairwise comparison matrix, and weight vector for all the criteria and subcriteria.

| $CR=\frac{CI}{RI}$ | ( 1 ) |
| --- | --- |
| $CI=\frac{\lambda-n}{n-1}$ | ( 2 ) |

Where $CI$ is the Consistency Index, $RI$ is the random consistency index, depending on the matrix order (n), and $\lambda$ is the eigenvalue (Saaty 1980).

| $P=\left[ \begin{matrix} \begin{matrix} 1 & l_{12} \\ l_{21} & 1 \end{matrix} & \begin{matrix} \cdots& l_{1n} \\ \cdots& l_{2n} \end{matrix} \\ \begin{matrix} \vdots& \vdots\\ l_{n1} & l_{n2} \end{matrix} & \begin{matrix} \ddots& \vdots\\ \cdots& 1 \end{matrix} \end{matrix} \right]=\left[ \begin{matrix} \begin{matrix} \left( \frac{w_{1}}{w_{1}} \right) & \left( \frac{w_{1}}{w_{2}} \right) \\ \left( \frac{w_{2}}{w_{1}} \right) & \left( \frac{w_{2}}{w_{2}} \right) \end{matrix} & \begin{matrix} \cdots& \left( \frac{w_{1}}{w_{n}} \right) \\ \cdots& \left( \frac{w_{2}}{w_{n}} \right) \end{matrix} \\ \begin{matrix} \vdots& \vdots\\ \left( \frac{w_{n}}{w_{1}} \right) & \left( \frac{w_{n}}{w_{2}} \right) \end{matrix} & \begin{matrix} \ddots& \vdots\\ \cdots& \left( \frac{w_{n}}{w_{n}} \right) \end{matrix} \end{matrix} \right]$ | ( 3 ) |
| --- | --- |
| $W=\left[ w_{1},w_{2},\ldots,w_{n} \right]$ | ( 4 ) |

**Appendix B.** Household Survey on Rainwater Harvesting

Good morning/afternoon. We are students from Universidad Industrial de Santander. We are conducting a household survey regarding rainwater harvesting systems (RWHS) in Garbanzal village (Los Santos municipality). The objective of this survey is to establish water uses and demand in the houses of this village, collect information regarding existing RWHS, and identify potential improvements.

**Presentation of the interviewer**

Your answers improve our knowledge regarding RWH, and our research results will be shared with the community through meetings and material such as leaflets or didactic booklets. If you have any questions regarding the project, please do not hesitate to ask them during the survey.

Your participation in the survey is voluntary. If you feel uncomfortable with any questions, you can tell the interviewer and not answer. The information you provided to the interviewer will be used only for this research and accessible only to the researchers linked to the project. The results could be published in academic journals or be presented in meetings, conferences, and other divulgation scenarios. These results would be aggregated for the whole village and not presented as individual responses. In all these results, your identity will be protected according to your preferences registered in the informed consent. The survey will extend for around 30 minutes. We appreciate your participation.

# Sociodemographic information (household head)

## Name: ______________________________________________________________

## Sex: F M

|

|

## Age: _____________

## Civil status: a. Single b. Married c. Free union d. Separated e. Divorced f. f. Widowed

|

|

|

|

|

|

## ¿ How long have you lived in this house?

- a. Since birth
- b. One year or less, state number of months: ______
- c. More than one year, state number of months: ______

## Current occupation

a. Farmer b. Day-laborer c. Merchant d. Public employee e. Retired f. Other, ¿Which? ______________________

|

|

|

|

|

|

## Educational level:

- a. Incomplete primary school, ¿how many years?_________
- b. Complete primary school
- c. Incomplete high school, ¿how many years? _________
- d. Complete high school
- f. Technical training
- g. Technological training
- h. Professional
- i. Other, ¿Which? ________________________________

## ¿ Do you belong to the community board? No Yes

|

|

## ¿Do you attend community meetings? No Yes

|

|

## ¿Anyone in your family belong to the community board? No Yes, ¿Who? ____________________________

|

|

## ¿ Anyone in your family attend community meetings? No Yes, ¿Who? ______________________________

|

|

## ¿ Do you belong to any community organization?

No Yes, ¿Which? ______________________________________________________________

|

|

## ¿ Anyone in your family belong to any community organization?

No Yes, ¿Who? __________________________________________________

|

|

## If yes, name the organization ______________________________________________________________________

## SOCIOECONOMIC INFORMATION (household inhabitants)

| **No.** | **Name (optional)** | **Relationship to the Head of Household** | **Age** | **Civil status** | **Occupation** | **Educational level** |
| --- | --- | --- | --- | --- | --- | --- |
| 1 |  |  |  |  |  |  |
| 2 |  |  |  |  |  |  |
| 3 |  |  |  |  |  |  |
| 4 |  |  |  |  |  |  |
| 5 |  |  |  |  |  |  |
| 6 |  |  |  |  |  |  |
| 7 |  |  |  |  |  |  |
| 8 |  |  |  |  |  |  |

# Household characteristics

## This farm is:

a. Owned b. Rented c. Sharecropping d. Borowed e. Family f. Other, ¿Which? ______________________

|

|

|

|

|

|

## ¿ What is the total area of this farm? __________________ Do not know not answer

|

## Household type (Observation)

a. House b. Shack c. Other, ¿Which? _________________________

|

|

|

## ¿ How many floors or levels does the house have?? ________________

## ¿ Does the house currently have gutters and downspouts (to collect water that runs off the roof)?? Yes No

|

|

*(If the answer is No, continue with question 2.11; otherwise, continue with question 2.6)*

## 2.6. Fill out the following table about gutters and downspouts.

*Include diameter where applicable. If not applicable or it is an open channel, fill in the height and width boxes.*

|  | **Diameter** | **Height** | **Width** | **Length** | **Material** | **Condition** |
| --- | --- | --- | --- | --- | --- | --- |
| Gutters |  |  |  |  | __a. Galvanized steel  __b. Stainless steel  __c. Aluminium  __d. Plastic  __e. Other, ¿Which?  **____________________**  __f. Do not know, not answer | __1. Very bad  __2. Bad  __3. Fair  __4. Good  __5. Excellent  __0. Do not know, not answer |
| Downspouts |  |  |  |  | __a. Acero galvanizado  __b. Acero inoxidable  __c. Aluminio  __d. Plástico  __e. Otro, ¿Cuál?  **____________________**  __f. Do not know, not answer | __1. Very bad  __2. Bad  __3. Fair  __4. Good  __5. Excellent  __0. Do not know, not answer |

^1^1. Very bad, require change; 2. Bad, broken in some parts; 3. Fair, it has some corrosion; 4. Good, needs cleaning; 5. Excellent, new.

## ¿ Is there any mesh to prevent solids from entering the system?? Yes No

|

|

## If the answer was yes, which components are there mesh? *(several options are possible)*

- Gutters
- Downspouts
- Tanks
- Other, ¿Which? ________________________________

## ¿ How many times a year do you perform maintenance work on the gutters and downspouts? ______________

## What do the maintenance tasks carried out on the gutters consist of? Briefly describe

**___________________________________________________________________________________________________________________**

**2.10. What are the maintenance tasks carried out on the downspouts? Briefly describe ___________________________________________________________________________________________________________________**

**What is the roof material of your house?**

a. Clay tile b. Straw c. Zinc sheet d. Cement tile e. Other: ______________

|

|

|

|

|

Do not know, not answer

|

## ¿What is the house roof area? ________ m^2^ Do not know not answer

|

## *(Observation)* ¿ How many slopes does the roof have? ___________

## ¿ How many times a year do you perform maintenance/cleaning work on the roof? _________

*(if the answer is no maintenance is performed, continue to question 2.16, otherwise continue to 2.15)*

## ¿ What does the maintenance you perform consist of? Briefly describe

**____________________________________________________________________________________________________________________**

## ¿ Do you use any type of waterproofing on the roof of your home?? Yes No

|

|

## ¿ Which of the following services does your house have?

a. Electric energy supply b. Water supply c. Wastewater collection d. Gas e. Telephone f. Internet g. None

|

|

|

|

|

|

|

## If you have electricity, is the supply constant throughout the year? Yes No

|

|

## **If the answer is no, how many days and hours per week do you have this service?** _______ days and _______ hours

# Information about water uses and demand

## I will ask you some questions about available water sources.

| **Water Source** | **What water sources do you use in your house? Mark with X** | **¿** **How do you access these sources? ^1^** | **Do you have a water meter for sources with house connections? Yes or No** | **How long does bringing the water from sources without a household connection take? minutes** | **How far from the house is the source for sources without household connection? Meters** | **For sources without household connections, ¿Who collects the water?** | **¿Is the source available all year round? Yes or NO** | **¿** **What months of the year is this water source not available?^2^** | **How many liters of water do you carry on each trip for sources without household connection?** | **Do you treat the water before its use?** |
| --- | --- | --- | --- | --- | --- | --- | --- | --- | --- | --- |
| Rainwater harvesting |  |  |  |  |  |  |  |  |  |  |
| Communal water supply from groundwater |  |  |  |  |  |  |  |  |  |  |
| Communal water supply from surface source |  |  |  |  |  |  |  |  |  |  |
| Water truck |  |  |  |  |  |  |  |  |  |  |
| Other, ¿which?  ______________ |  |  |  |  |  |  |  |  |  |  |

^1^ a) household connection; b) without household connection; c) Other, ¿which?; ^2^Includes months as numbers; ^3^a) Sedimentation, b) Filtration, c) Chlorine, d) Boling, e) Other, ¿which?, f) Do not know not answer, g) None.

## Do you buy water in a bottle/bag? Yes No

|

|

*(If the answer is Yes, continue with question 3.3; if the answer is No, go to question 3.7)*

## If the answer is yes, how often do you buy bottled/bag water for your house? (Fill out only one of the options depending on the frequency)

_______ times per day; _______ times per week; _______ times per month; _______ times per year

**How many liters of water do you buy each time you need it?**_______________________

## How much is the cost of the purchased water?___________________________

## What use do you give to the bottled/bag water you buy? ___ Drinking ___ Cooking ___Other, ¿Which? _____________________

## If your house has access to the communal water source (question 3.1), How much do you pay annually for this service? ______________________

## If your house has access to the communal groundwater source (question 3.1), How much do you pay annually for this service? ______________________

## ¿ Has it happened in the last month that your home did not have enough water when you needed it?

Yes No

*(If the answer is No, go to question 3.12; if the answer is Yes, continue with 3.10)*

## ¿ How many days was there no water supply? __________

## ¿ Which activities did not have a water supply on those days?

________________________________________________________________________________________________________________

## ¿ Do you have a garden in your home?? No Yes

|

|

## If you have a garden, ¿ What is the irrigation frequency in the dry season? _______ per day or ________ per week

## ¿ Do you have crops in your plot? No Yes

|

|

*(If the answer is yes, fill out the table in question 3.16; if the answer is no, go to question 3.17)*

## 3.14. Answer the following questions about the crops you have.

| **¿What crops do you have?** | **What area is available for each crop? (m^2^)** | **For what purposes do you have this crop?^1^** | **What water source do you use for each crop?** | **In what months do you irrigate each crop?^2^** | **How often do you irrigate the crops in these months?** | **¿Cuánto tiempo dura regando los cultivos?** | **¿Qué tipo de riego utiliza?^3^** |
| --- | --- | --- | --- | --- | --- | --- | --- |
|  |  |  |  |  | ____ times per day  ____ times per week | ____ mins  ____ hours |  |
|  |  |  |  |  | ____ times per day  ____ times per week | ____ mins  ____ hours |  |
|  |  |  |  |  | ____ times per day  ____ times per week | ____ mins  ____ hours |  |
|  |  |  |  |  | ____ times per day  ____ times per week | ____ mins  ____ hours |  |
|  |  |  |  |  | ____ times per day  ____ times per week | ____ mins  ____ hours |  |
|  |  |  |  |  | ____ times per day  ____ times per week | ____ mins  ____ hours |  |

^1^1 Self-consumption or 2 sell; ^2^ Write the months in number format; ^3^1 Surface, 2 sprinkler, 3 drip, 4 sub, 5 other, which.

## ¿ Do you raise animals on your farm? No Yes

|

|

*(If the answer is yes, fill out the table in question 3.18; if the answer is no, go to question 3.19)*

## Fill out the following table about the animals you raise.

| **Type** | **¿Which animals do you raise? Mark with an X** | **Quantity** | **¿** **What area is available for each group of animals? ^a^ (m2)** | **¿** **For what purposes do you raise these animals? ^b^** | **What water source do you use to feed the animals?** | **How many times a day do you water the animals?** | **How much water do you estimate you use each time you water the animals? (liters)** |
| --- | --- | --- | --- | --- | --- | --- | --- |
| Birds (chickens, ducks, etc.) |  |  |  |  |  |  |  |
| Cows |  |  |  |  |  |  |  |
| Horses |  |  |  |  |  |  |  |
| Pigs |  |  |  |  |  |  |  |
| Sheep |  |  |  |  |  |  |  |
| Goats |  |  |  |  |  |  |  |
| Other: |  |  |  |  |  |  |  |

^a^ Only if you have them in a fenced area; ^b^ 1 Self-consumption or 2 sell;

## ¿In what months do you believe is the dry season? __________________________________________________

## ¿ In what months do you believe is the wet season? ________________________________________________

## How often do you think it rains in the village during the rainy season?

a. Everyday b. Twice a week c. Once a week d. Other, ¿Which? _____________________

|

|

|

|

## ¿ Does your home have a tank where you store water that does not come from rain? Yes No

*(If yes, continue with question 3.23, otherwise skip to section 4)*

## List each tank you have (do not include rainwater tanks because there is a specific section for these)

| **No** | **Water source to fill this tank** | **Purpose of the collected water** | **Capacity (L)** | **Material** | **Frequency of cleaning and maintenance ^2^** |
| --- | --- | --- | --- | --- | --- |
| 1 |  |  |  |  |  |
| 2 |  |  |  |  |  |
| 3 |  |  |  |  |  |
| 4 |  |  |  |  |  |
| 5 |  |  |  |  |  |

## Describe the maintenance tasks developed __________________________________________________________________________________________________________________

# Information on perception related to rainwater

## ¿ Do you think the inhabitants of the village suffer from water scarcity? Yes No

*(If the answer is Yes, continue with question 4.2; if the answer is No, go to question 4.7)*

## ¿ How does this scarcity problem affect you? __________________________________________________________________

## ¿ Does the water scarcity prevent you from carrying out any activity? Yes No

## ¿ What is the activity that you cannot do? ___________________________________________________________________________

## ¿ Do you think that rainwater harvesting is a good alternative to the problem of water scarcity? Yes No

## ¿Why?___________________________________________________________________________________________________

## On a scale of 1 to 5, how clean do you think the rainwater is, where 1 is very dirty water, and 5 is very clean water

1(very dirty) 2(Dirty) 3(Fairly clean) 4(Clean) 5(Very clean)

## ¿ What possible risks do you think the use of rainwater entails?

- a. Stomach diseases (associated with drinking it)
- b. Skin diseases (irritation due to direct contact with the skin)
- c. Skin diseases (irritation from contact with clothes washed in rainwater)
- d. Damage to clothing from washing using rainwater
- e. Other, ¿Which? ______________________________________________________________________________________________

## On a scale of 1 to 5, how useful do you perceive the use of rainwater harvesting systems?

1(Useless) 2(little useful) 3(Moderately useful) 4(Useful) 5(Very useful)

## ¿ On a scale of 1 to 5, how easy is it to use a rainwater harvesting system?

1(Very difficult) 2(Difficult) 3(Fair) 4(Easy) 5(Very easy)

## Do you know what components a rainwater harvesting system has? No Yes, explain:

___________________________________________________________________________________________________________________

___________________________________________________________________________________________________________________

## ¿ Do you know how a rainwater harvesting system works? No Yes, explain:

___________________________________________________________________________________________________________________

___________________________________________________________________________________________________________________

## ¿ Do you know how to install a rainwater harvesting system? No Yes, explain:

___________________________________________________________________________________________________________________

___________________________________________________________________________________________________________________

## ¿ Do you know what maintenance activities should occur on rainwater harvesting systems? No Yes, ex plain:

___________________________________________________________________________________________________________________

___________________________________________________________________________________________________________________

# Information on existing rainwater harvesting systems

# *(only to those that already have rainwater harvesting systems)*

## ¿ Does your home have tanks for rainwater collection? Yes No

## ¿ How many rainwater collection tanks do you have? ________________

## Fill out the following table about existing rainwater collection tanks.

| **No.** | **How many liters does this tank store?** | **What material is this tank made of?** | **How many times do you perform cleaning/ maintenance per year?** | **What condition do you think the tank is in?** | **Apart from the tank, do you have any other components or accessories?** | **How much do you estimate this system cost?** | **What roof area drains into this tank?** | **What material is the roof that drains water for this tank?** | **How long have you had this tank?** |
| --- | --- | --- | --- | --- | --- | --- | --- | --- | --- |
| 1 |  |  |  |  |  |  |  |  | _____ Years o  _____ Months |
| 2 |  |  |  |  |  |  |  |  | _____ Years o  _____ Months |
| 3 |  |  |  |  |  |  |  |  | _____ Years o  _____ Months |
| 4 |  |  |  |  |  |  |  |  | _____ Years o  _____ Months |
| 5 |  |  |  |  |  |  |  |  | _____ Years o  _____ Months |
| 6 |  |  |  |  |  |  |  |  | _____ Years o  _____ Months |
| 7 |  |  |  |  |  |  |  |  | _____ Years o  _____ Months |
| 8 |  |  |  |  |  |  |  |  | _____ Years o  _____ Months |

## What do the cleaning/maintenance tasks you perform consist of? Briefly describe

________________________________________________________________________________________________________________

## Regarding the amount of rainwater collected in your system, you consider that:

- a. You are satisfied with the quantity
- b. You are satisfied with the quantity, but you would like to increase the quantity
- c. If you are not satisfied with the quantity, you need to increase the quantity

## ¿ What is the use of rainwater collected in the storage tank?

| **Tank** | **Use** | **Tank** | **Use** |
| --- | --- | --- | --- |
|  | Drinking |  | Watering animals |
|  | Cooking |  | Cleaning of the household |
|  | Washing dishes |  | Cleaning of the car |
|  | Showering |  | Flushing toilets |
|  | Hand washing |  | Irrigation of crops |
|  | Cleaning of clothes |  | Cleaning of stables |

## ¿ Is buying sodium hypochlorite (Chlorine) in your area easy? Yes No

## ¿ What benefits did the installation of the rainwater harvesting system bring to you and your family?

____________________________________________________________________________________________________________________

____________________________________________________________________________________________________________________

# Information about rainwater systems *(Only if people lack a rainwater harvesting system)*

## ¿ Do you have enough area on your farm to install a rainwater harvesting system? Yes No

## ¿ Is buying sodium hypochlorite (Chlorine) in your area easy? Yes No

## ¿ Would you be willing to use rainwater in your house? Yes No

## ¿ Would you like to implement a rainwater harvesting system in your house? Yes No

*(If the answer is Yes, continue with question 6.5. If the answer is No, go to question 6.8)*

## If the answer is yes, why?

- a. Saving money on water purchases
- b. Good for the environment
- c. Increase the available water
- e. Save time and effort carrying water from water sources nearby
- Other: ___________________________________________________________________

## If yes, what use would you give to the collected rainwater? *(Mark with an X the options that apply)*

| **Marque x** | **Use** | **Marque x** | **Use** |
| --- | --- | --- | --- |
|  | Drinking |  | Watering animals |
|  | Cooking |  | Cleaning of the household |
|  | Washing dishes |  | Cleaning of the car |
|  | Showering |  | Flushing toilets |
|  | Hand washing |  | Irrigation of crops |
|  | Cleaning of clothes |  | Cleaning of stables |
|  | Drinking |  | Watering animals |

## ¿ How much money would you invest in building a rainwater collection system?

- a. 500.000 - 1.000.000
- b. 1.000.000 - 2.000.000
- c. 2.000.000 - 3.000.000
- d. 3.000.000 - 5.000.000
- e. > 5.000.000

## If you are NOT willing to invest in building a collection system, why?

- a. High investment cost
- b. Is not willing to undertake operation and maintenance activities
- c. Lack of knowledge about system installation, operation, or maintenance
- d. Other: _______________________________________________________________________

## what would motivate you to install a rainwater collection system if you are not willing to invest in building a collection system?

- a. Low-interest loans for installation
- b. Government economic subsidies (total value of investment)
- c. Government economic subsidies (partial value of the investment)
- d. Delivery of materials necessary for the construction of the system
- e. training on the installation, operation, and operation of the system
- f. Policies or laws that make the installation of these systems mandatory
- g. Other, ¿Which? _____________________________________________________________

## ¿ What benefits do you think installing a rainwater harvesting system could bring you and your family??

____________________________________________________________________________________________________________________

____________________________________________________________________________________________________________________

**¡** **The information you provide is essential to us. Thank you very much for your time and willingness to answer this survey!**

**SUPPLEMENTARY SURVEY**

This section investigates your opinion on the available water sources in the village.

Name: ________________________________________________________________________________

Telephone*: __________________________

* It is not mandatory; only if you want us to call you to let you know when we will return to share the survey results.

**1. INFORMATION REGARDING WATER COSTS**

This section inquires about the costs of having water in your house.

**1. Fill out the information in the following table about the costs of the sources available in the area. If you have a source other than those mentioned in the table, fill in the box "Other, which one?"**

| **Water source** | **1.1. ¿** **Do you have to pay to use water from this source?**  **Yes or no** | **1.2. ¿** **How often do you pay to use water from this source?^1^** | **1.3. ¿** **How many liters do you buy or pay for each time you use water from this source?** | **1.4. ¿** **How much does the amount of water you indicated in question 1.3 cost?** |
| --- | --- | --- | --- | --- |
| a. Bottled water |  |  |  |  |
| b. Community water supply system (groundwater) |  |  |  |  |
| c. Community water supply system (surface water) |  |  |  |  |
| d. Water truck |  |  |  |  |
| e. Other, ¿Which?: |  |  |  |  |

^1^Annual frequency

**2. INFORMATION ABOUT CROPS**

This section inquires about your crops.

**2.1. ¿Do you have maize crops in your plot?** Yes No

*(If the answer is yes, answer question 2.2. if the answer is no, continue to section 3.)*

**2.2. ¿How many square meters do you have with maize crops? Or ¿How many plants do you have?**

a. Area: __________

b. Plants: ___________

**2.3. ¿What are the purposes of your maize crops?** *(Mark the answer with an X; you can mark several options if required.)*

__ a. Self-consumption for the family

__ b. Self-consumption for feeding animals.

__ c. Sell

__ d. Other, ¿Which? _____________________________________________________________

**2.4. The following table records information about planting, irrigation, harvesting, and the products used in growing maize.**

Mark with an X for the month you develop activities such as sowing, watering, or harvesting, and the months you use products such as fertilizers, herbicides, or other products to control plagues and diseases. Leave the boxes blank if you do not water or use products in the crop. If you apply the products in items b, d, e, f, or g more than once during the month, write down in the box how many times you do it during the month.

|  | **Jan** | **Feb** | **Mar** | **Apr** | **May** | **Jun** | **Jul** | **Aug** | **Sep** | **Oct** | **Nov** | **Dec** |
| --- | --- | --- | --- | --- | --- | --- | --- | --- | --- | --- | --- | --- |
| a. Sowing |  |  |  |  |  |  |  |  |  |  |  |  |
| b. Watering |  |  |  |  |  |  |  |  |  |  |  |  |
| c. Harvesting |  |  |  |  |  |  |  |  |  |  |  |  |
| d. Using fertilizasers |  |  |  |  |  |  |  |  |  |  |  |  |
| e. Using herbicides |  |  |  |  |  |  |  |  |  |  |  |  |
| f. Using products for controlling plagues and diseases |  |  |  |  |  |  |  |  |  |  |  |  |
| g. Other |  |  |  |  |  |  |  |  |  |  |  |  |

**2.5. If you use any of the products in items d, e, f, or g of question 2.4. Which product do you use in each case?** *Note N.A., which you do not use.*

d. Fertilizers _________________

e. Herbicides _________________

f. Products for controlling plagues and diseases _______________

g. Other, ¿Which? ___________________

**2.6. If you use any of the products in items d, e, f, or g of question 2.4, ¿How much product do you use per harvest?** *Note N.A. in, which you do not use.*

d. Fertilizers _________________

e. Herbicides _________________

f. Products for controlling plagues and diseases _______________

g. Other, ¿Which? ___________________

**2.7. If you use any of the products in items d, e, f, or g of question 2.4, ¿** **How much does the quantity of product that you indicated in question 2.6 cost?** *Note N.A., which you do not use.*

d. Fertilizers _________________

e. Herbicides _________________

f. Products for controlling plagues and diseases _______________

g. Other, ¿Which? ___________________

**2.8. ¿Do you irrigate your crop?** Yes No

*(If the answer is yes, answer question 2.9. if the answer is no, continue to question 2.10.)*

**2.9. ¿** **How many liters of water do you use each time you water your crop?** (consider the entire area indicated in question 2.2) _____________________

**2.10. ¿** **How many kg of maize does your crop produce per harvest?** __________________

**2.11. ¿** **Is it common for pests or diseases to occur in crops in the area?** Yes No

*(If the answer is yes, answer question 2.12., if the answer is no, continue to section 3)*

**2.12. ¿** **What pests and diseases occur?** ________________________________________________

**3. OCASIONAL WATER USES**

In this section, we are interested in knowing about the activities in which you use water occasionally, that is, uses that are not daily (e.g., washing clothes, general cleaning of the home, etc.)

**3.1. Mark with an X the day or days the following activities are carried out. If they are carried out less frequently than weekly (e.g., biweekly, monthly, etc.), fill in the frequency in the box other^2^.**

| **Activities** | **M** | **T** | **W** | **T** | **F** | **S** | **S** | **Other** |
| --- | --- | --- | --- | --- | --- | --- | --- | --- |
| Washing clothes |  |  |  |  |  |  |  |  |
| Cleaning the house |  |  |  |  |  |  |  |  |
| Washing the car |  |  |  |  |  |  |  |  |
| Cleaning stables |  |  |  |  |  |  |  |  |
| Watering plants or gardens |  |  |  |  |  |  |  |  |
|  |  |  |  |  |  |  |  |  |
|  |  |  |  |  |  |  |  |  |

**4. EVALUATION OF SUBCRITERIA**

**Social subcriteria: Perceived ease of use (S.1)**

To answer questions 4.1 to 4.4. Each alternative rainwater harvesting system will be shown and explained in a diagram.

**4.1. ¿** **How difficult is it to use the first rainwater harvesting system shown?**

| □ Very difficult | □ Difficult | □ Fair | □ Easy | □ Very easy |
| --- | --- | --- | --- | --- |

**4.2. ¿** **How difficult is it to use the second rainwater harvesting system shown?**

| □ Very difficult | □ Difficult | □ Fair | □ Easy | □ Very easy |
| --- | --- | --- | --- | --- |

**4.3. ¿** **How difficult is it to use the third rainwater harvesting system shown?**

| □ Very difficult | □ Difficult | □ Fair | □ Easy | □ Very easy |
| --- | --- | --- | --- | --- |

**Social subcriteria: Available water sources (S.2)**

**4.4. Regarding the amount of water you have available from sources other than rainwater, you consider that the amount is:**

| □ Very low | □ Low | □ Fair | □ High | □ Very high |
| --- | --- | --- | --- | --- |

**The information you provided is essential to us. Thank you very much for your time and willingness to answer this survey!**

**Appendix C.** Mass balance method THETA

| $Y_{t}=\min\left\{ \begin{matrix} D_{t} \\ V_{t-1}+\theta Q_{t} \end{matrix} \right.$ | ( 1 ) |
| --- | --- |
| $V_{t}=\min\left\{ \begin{matrix} \left( V_{t-1}+Q_{t}-\theta Y_{t} \right)-\left( 1-\theta\right)Y_{t} \\ S-\left( 1-\theta\right)Y_{t} \end{matrix} \right.$ | ( 2 ) |

Where $D_{t}$ is water demand (m^3^) during a time interval $t$, $V_{t}$ is the stored water volume (m^3^) during $t$, $\theta$ is a parameter between 0 and 1, $Q_{t}$ is the rainfall that runs on the roof during $t$, $Y_{t}$ is the storage yield (m^3^) during $t$, and $S$ is the storage capacity (m^3^).

**Appendix D.** Results of the process of screening subcriteria.

Subcriteria found

294

Filtered subcriteria:

76

First filter

Filtered subcriteria:

44

Second filter

Filtered subcriteria:

22

Third filter

Filtered subcriteria:

7

Fourth filter

| Economic: | 53 |
| --- | --- |
| Social: | 91 |
| Technical: | 150 |

| Economic: | 15 |
| --- | --- |
| Socials: | 8 |
| Technical: | 21 |

| Economic: | 15 |
| --- | --- |
| Social: | 40 |
| Technical: | 21 |

| Economic: | 7 |
| --- | --- |
| Social: | 4 |
| Technical: | 11 |

| Economic: | 2 |
| --- | --- |
| Social: | 2 |
| Technical: | 3 |

**Appendix E.** Scoring of items of the maintenance subcriteria.

Table E.1. Scoring of items of the maintenance subcriteria

| Item | Difficulty | Description |
| --- | --- | --- |
| M1. Tools | 1 | Not required |
|  | 2 | Typically found in the household |
|  | 3 | Must be purchased |
|  | 4 | Must be purchased, but they are difficult to acquire |
|  | 5 | Equipment or machinery must be rented |
| M2. Personal | 1 | Any person (system knowledge is not required) |
|  | 2 | Any person who has had contact with the system |
|  | 3 | The system user can do it |
|  | 4 | Qualified (The system builder) |
|  | 5 | Qualified (A qualified specialist must be trained) |
| M3. Annual Frequency | 1 | Less than once a year |
|  | 2 | 1 or 2 (biannual or annual) |
|  | 3 | 3 or 4 (quarterly or every four months) |
|  | 4 | Between 5 and 12 (between biannual and monthly) |
|  | 5 | 52 or more (weekly or more frequently) |
| M4. Materials (chlorine, detergent, pipe, channel, etc.) | 1 | Not required |
|  | 2 | Typically found in the household |
|  | 3 | Must be purchased |
|  | 4 | Must be purchased, but they are difficult to acquire |
|  | 5 | Not regionally available |

**Appendix F.** Annual cycle of rainfall in "La Mesa" station (1974 – 2021)

Fig. F1 Annual cycle of rainfall in "La Mesa" station (1974 – 2021)

**Appendix G.** Results of rainwater quality parameters assessed in Garbanzal

Table G.1. Results of rainwater quality parameters assessed in Garbanzal.

| **Parameter** | **N** | **Measured value** | **Standard** | |
| --- | --- | --- | --- | --- |
|  |  |  | **Minimum value^a^** | **Maximum value^a^** |
| Physicochemical |  |  |  |  |
| Turbidity [NTU] | 12 | 1.94 ± 1.31 | - | 5 |
| pH [unit. pH] | 12 | 7.51 ± 0.89 | 6.5 | 8.5 |
| Conductivity [μs/cm] | 12 | 70.40 ± 36.84 | N.E. ^b^ | N.E. ^b^ |
| Total iron [mg/L] | 5 | 0.054 ± 0.01 | - | 0.3 |
| Nitrates [mg/L] | 5 | 0.932 ± 0.98 | - | 50 |
| Total Zinc [mg/L] | 5 | 0.052 ± 0.06 | N.E. ^b^ | N.E. ^b^ |
| Residual Chlorine [mg/L] | 5 | Non-detectable | 0.2 | 5 |
| Apparent color [PCU] | 5 | 46 ± 26.57 | N.E. ^b^ | N.E. ^b^ |
| Microbiological |  |  |  |  |
| Total Coliforms [CFU/100 ml] | 5 | 1046 ± 1933 | - | 0 |
| E. coli [CFU/100 ml] | 14 | 33 ± 45 | - | 0 |

Note: ^a^The minimum and maximum values correspond to the WHO standard [64]. ^b^N.E. No acceptable maximum or minimum value is specified.

**Appendix H.** Efficiency versus volume for the three proposed alternatives.

Fig. H1 Efficiency versus volume graph for the three proposed alternatives

**Appendix I.** Components of each proposed Rainwater Harvesting System alternative.

The components of each proposed alternative are shown in Table D.1.

Table I.1. Description of the components of the three rainwater harvesting system alternatives.

| **Catchment area.** Considered as the household roof (Abdulla and Al-Shareef 2009). The roof area selected for the three alternatives was 100 m^2^ (Fig. 8a). Additionally, in the alternatives where small-scale productive water uses are included, it was assumed that people have a warehouse that provided an additional roof area (Fig. 8b). The material proposed for the three alternatives is sheet metal, typical in the study area and by recommendation of Abdulla & Al-Shareef (2009). | 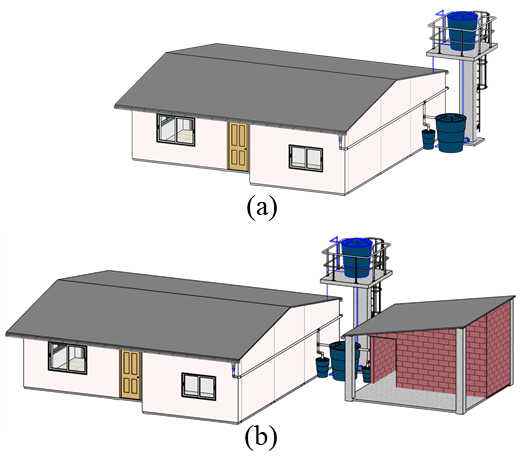  Fig. 8 Catchment area of alternatives. Source: Authors. |
| --- | --- |
| **Gutters and downpipes.** Gutters and downpipes were selected considering the commercially available materials for the three alternatives as recommended by Unatsabar (2001). The selected gutter was rectangular with a 12 cm base and 6 cm height (Fig. 9). This gutter size has hydraulic properties similar to the gutters suggested by Abdulla & Al-Shareef (2009). Downpipes were Policlorur Vynil of 3" to carry water from the household roof and 2" from the warehouse in alternatives 2 and 3. | 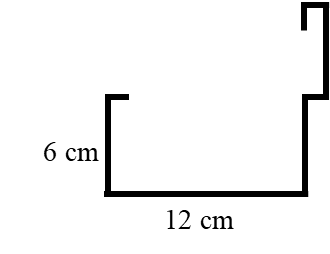  Fig. 9 Gutter transversal section. Source: Authors based on commercial section. |
| **Prefiltration.** Treatment to remove big solids and settleable particles by gravity without chemicals. Alternative 1 had mesh in the gutter (Fig. 10a), basket strainer in downpipes (Fig. 10b) and a plastic first-flush diverter of 110 L (Fig. 10c). Alternatives 2 and 3 had mesh in the gutter (Fig. 10a), basket strainer in downpipes (Fig. 10b) and two plastic first-flush diverters, uno de 110 L and other of 20 L (Fig. 10d). These two components are suggested by Mazurkiewicz et al. (2022) as the two first processes from four to produce potable water from rainwater. | 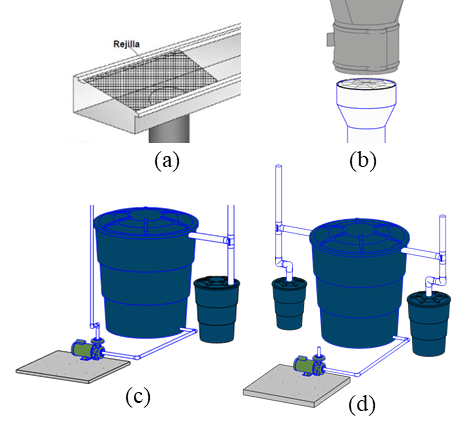  Fig. 10 Components of the primary treatment. Source: (a) Ávila (2013), (b) Authors based on CAWST. (2009), (c) Authors based on Unatsabar (2001), (d) Authors based on Unatsabar (2001). |
| **Storage tank.** In the area, most households had between 1 and 3 tanks, for which this study considered it feasible to implement 2 tanks of 1.30 m-height and an average diameter of 1.08 m. One tank is above ground to pump water for a minimum daily interval to an elevated storage tank (Fig. 11). This tank is connected to the household faucets and provides water by gravity. The tanks were plastic. The storage volume was calculated using mass balance methods at 2000L for the three alternatives, with an efficiency of 37%, 33%, and 40 % for alternatives 1, 2, and 3, respectively. | 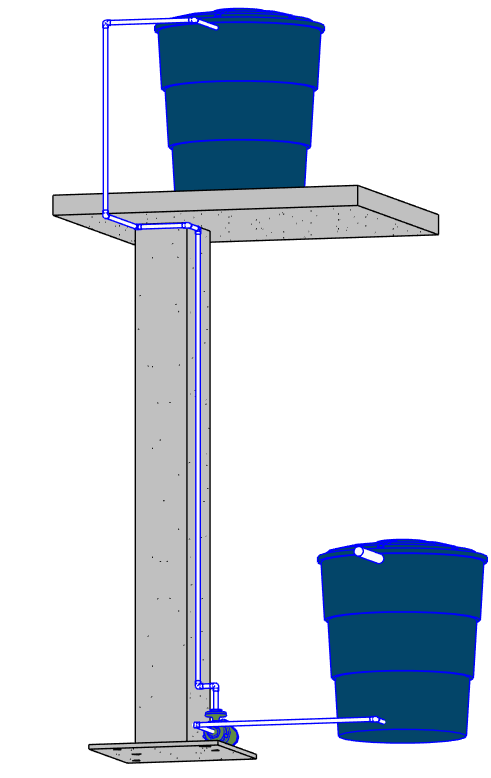  Fig. 11 Configuration of storage tanks. Source: authors. |
| **Distribution network.** It was assumed there were four sanitary devices: a washbasin, a toilet, a shower, and a kitchen faucet (Fig. 12a). To adequately distribute water by gravity, the level in the elevated storage tank must be 4.3 m in the three alternatives. For this, a ½ hp pump works with an efficiency of 80% during 15 minutes daily (Fig. 12b). | 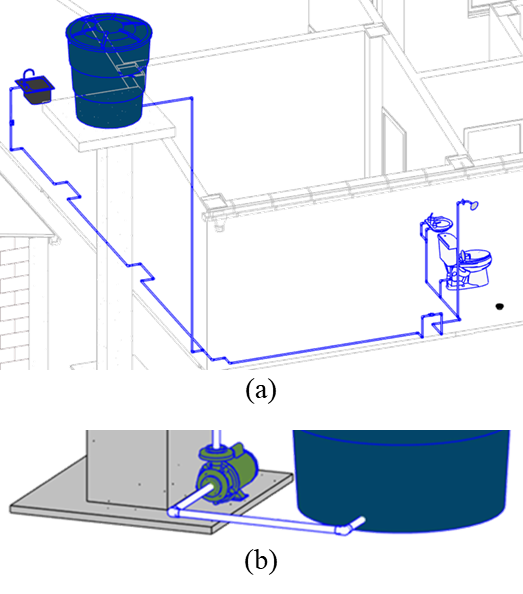  Fig. 12 Rainwater distribution network. Source: Authors |
| **Filtration.** A filter built inside a Polyvinylchloride of 6", minimum diameter proposed by CAWST (2009), with a filtration velocity of 0.4 m/h (Fig. 13), maximum volume that could be used in slow sand filtration according to Ramírez & Pérez (2002). | 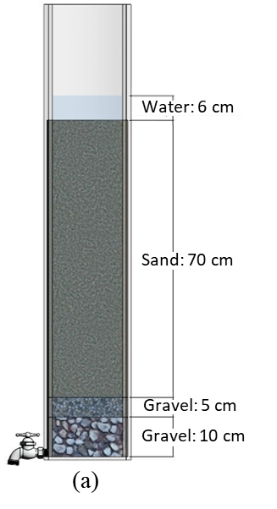  Fig. 13 Slow sand filter. Source: Authors based on Posadas (2015). |
| **Physical disinfection by boiling.** The most simple disinfection method is available for all people in the study area since it is a physical disinfection method that can be used at the household scale and eliminate any pathogen microorganism present in water (Nossa Fuentes 2017). |  |

**Appendix J.** Cashflow for the estimation of cost and benefits discounted by each alternative.

Table J.1. Cashflow for the estimation of cost and benefits discounted by Alternative 1.

| Alternative 1 |  |  |  | **Net cash flow** |
| --- | --- | --- | --- | --- |
| **Date** | **Benefit of water savings** | **Maintenance** | **Operation** | -$ 4.962.384 |
| dic-23 | $ 248.756 | $ 56.710 | $ 31.808 | $ 160.239 |
| dic-24 | $ 259.002 | $ 46.443 | $ 33.118 | $ 179.440 |
| dic-25 | $ 269.669 | $ 38.386 | $ 34.482 | $ 196.800 |
| dic-26 | $ 280.776 | $ 50.348 | $ 35.903 | $ 194.526 |
| dic-27 | $ 292.340 | $ 81.426 | $ 37.381 | $ 173.533 |
| dic-28 | $ 304.381 | $ 54.580 | $ 38.921 | $ 210.879 |
| dic-29 | $ 316.917 | $ 45.112 | $ 40.524 | $ 231.281 |
| dic-30 | $ 329.970 | $ 59.169 | $ 42.193 | $ 228.608 |
| dic-31 | $ 343.560 | $ 48.904 | $ 43.931 | $ 250.725 |
| dic-32 | $ 357.710 | $ 254.525 | $ 45.740 | $ 57.445 |
| dic-33 | $ 372.443 | $ 53.016 | $ 47.624 | $ 271.803 |
| dic-34 | $ 387.782 | $ 70.741 | $ 49.585 | $ 267.456 |
| dic-35 | $ 403.754 | $ 790.332 | $ 51.628 | -$ 438.206 |
| dic-36 | $ 420.383 | $ 75.382 | $ 53.754 | $ 291.247 |
| dic-37 | $ 437.697 | $ 121.912 | $ 55.968 | $ 259.817 |
| dic-38 | $ 455.724 | $ 81.719 | $ 58.273 | $ 315.732 |
| dic-39 | $ 474.494 | $ 67.543 | $ 60.673 | $ 346.279 |
| dic-40 | $ 494.037 | $ 88.589 | $ 63.172 | $ 342.276 |
| dic-41 | $ 514.385 | $ 73.221 | $ 65.774 | $ 375.390 |
| dic-42 | $ 535.570 | $ 381.079 | $ 68.483 | $ 86.008 |
| dic-43 | $ 557.629 | $ 79.376 | $ 71.303 | $ 406.949 |
| dic-44 | $ 580.595 | $ 104.110 | $ 74.240 | $ 402.245 |
| dic-45 | $ 604.508 | $ 86.050 | $ 77.298 | $ 441.161 |
| dic-46 | $ 629.406 | $ 112.863 | $ 80.482 | $ 436.061 |
| dic-47 | $ 655.329 | $ 182.529 | $ 83.796 | $ 389.004 |
|  |  |  |  |  |
| **Present value** | $ 6.460.009 | $ 1.909.239 | $ 826.035 |  |
| **Total discounted benefits** | $ 6.460.009 |  |  |  |
| **Total discounted costs** | $ 7.697.658 |  |  |  |

Table J.2. Cashflow for the estimation of cost and benefits discounted by Alternative 2.

| **Alternative 2** |  |  |  |  |  |  |  | **Net cash flow** |
| --- | --- | --- | --- | --- | --- | --- | --- | --- |
| **Date** | **Benefit of water savings** | **Benefit of water** | **Costs animals** | **Benefits crops** | **Costs crops** | **Maintenance** | **Operation** | -$ 5.337.149 |
| dic-23 | $ 134.582 | $ 1.793.600 | $ 1.485.485 | $ 171.859 | $ 221.400 | $ 57.279 | $ 31.808 | $ 304.068 |
| dic-24 | $ 140.125 | $ 2.544.035 | $ 1.467.756 | $ 178.937 | $ 275.502 | $ 47.036 | $ 33.118 | $ 1.039.685 |
| dic-25 | $ 145.896 | $ 2.648.815 | $ 1.528.207 | $ 186.307 | $ 286.848 | $ 39.004 | $ 34.482 | $ 1.092.476 |
| dic-26 | $ 151.905 | $ 2.757.911 | $ 1.591.149 | $ 193.980 | $ 298.663 | $ 50.991 | $ 35.903 | $ 1.127.091 |
| dic-27 | $ 158.161 | $ 2.871.500 | $ 1.901.405 | $ 201.970 | $ 310.964 | $ 82.335 | $ 37.381 | $ 899.546 |
| dic-28 | $ 164.675 | $ 2.989.767 | $ 1.724.916 | $ 210.288 | $ 323.771 | $ 55.278 | $ 38.921 | $ 1.221.845 |
| dic-29 | $ 171.458 | $ 3.112.905 | $ 1.795.959 | $ 218.949 | $ 337.106 | $ 45.838 | $ 40.524 | $ 1.283.885 |
| dic-30 | $ 178.520 | $ 3.241.115 | $ 1.869.929 | $ 227.967 | $ 350.990 | $ 59.925 | $ 42.193 | $ 1.324.564 |
| dic-31 | $ 185.872 | $ 3.374.605 | $ 1.946.944 | $ 237.356 | $ 365.447 | $ 49.691 | $ 43.931 | $ 1.391.820 |
| dic-32 | $ 193.528 | $ 3.513.593 | $ 2.326.576 | $ 247.132 | $ 380.498 | $ 291.054 | $ 45.740 | $ 910.384 |
| dic-33 | $ 201.498 | $ 3.658.306 | $ 2.110.623 | $ 257.311 | $ 396.169 | $ 53.869 | $ 47.624 | $ 1.508.830 |
| dic-34 | $ 209.797 | $ 3.808.979 | $ 2.197.552 | $ 267.908 | $ 412.486 | $ 71.629 | $ 49.585 | $ 1.555.432 |
| dic-35 | $ 218.438 | $ 3.965.857 | $ 2.288.062 | $ 278.943 | $ 429.475 | $ 956.980 | $ 51.628 | $ 737.093 |
| dic-36 | $ 227.435 | $ 4.129.197 | $ 2.382.299 | $ 290.431 | $ 447.164 | $ 76.344 | $ 53.754 | $ 1.687.502 |
| dic-37 | $ 236.802 | $ 4.299.265 | $ 2.846.820 | $ 302.393 | $ 465.581 | $ 123.274 | $ 55.968 | $ 1.346.818 |
| dic-38 | $ 246.555 | $ 4.476.337 | $ 2.582.578 | $ 314.848 | $ 484.756 | $ 82.763 | $ 58.273 | $ 1.829.370 |
| dic-39 | $ 256.710 | $ 4.660.701 | $ 2.688.945 | $ 327.815 | $ 504.722 | $ 68.629 | $ 60.673 | $ 1.922.257 |
| dic-40 | $ 267.283 | $ 4.852.660 | $ 2.799.693 | $ 341.317 | $ 525.510 | $ 89.720 | $ 63.172 | $ 1.983.164 |
| dic-41 | $ 278.291 | $ 5.052.524 | $ 2.915.003 | $ 355.374 | $ 547.154 | $ 74.399 | $ 65.774 | $ 2.083.860 |
| dic-42 | $ 289.753 | $ 5.260.620 | $ 3.483.395 | $ 370.011 | $ 569.689 | $ 435.772 | $ 68.483 | $ 1.363.045 |
| dic-43 | $ 301.687 | $ 5.477.287 | $ 3.160.066 | $ 385.250 | $ 593.152 | $ 80.653 | $ 71.303 | $ 2.259.049 |
| dic-44 | $ 314.113 | $ 5.702.877 | $ 3.290.218 | $ 401.118 | $ 617.582 | $ 105.440 | $ 74.240 | $ 2.330.627 |
| dic-45 | $ 327.050 | $ 5.937.759 | $ 3.425.730 | $ 417.638 | $ 643.018 | $ 87.434 | $ 77.298 | $ 2.448.966 |
| dic-46 | $ 340.520 | $ 6.182.314 | $ 3.566.824 | $ 434.839 | $ 669.502 | $ 114.304 | $ 80.482 | $ 2.526.561 |
| dic-47 | $ 354.545 | $ 6.436.943 | $ 4.262.314 | $ 452.749 | $ 697.077 | $ 184.568 | $ 83.796 | $ 2.016.481 |
|  |  |  |  |  |  |  |  |  |
| **Present value** | $ 3.494.982 | $ 62.825.373 | $ 37.776.397 | $ 4.463.044 | $ 6.829.803 | $ 2.082.827 | $ 826.035 |  |
| **Total discounted benefits** | $ 70.783.399 |  |  |  |  |  |  |  |
| **Total discounted costs** | $ 52.852.212 |  |  |  |  |  |  |  |

Table J.3. Cashflow for the estimation of cost and benefits discounted by Alternative 3.

| **Alternative 3** |  |  |  |  |  | **Net cash flow** |
| --- | --- | --- | --- | --- | --- | --- |
| **Date** | **Benefits of water savings** | **Benefit of animals** | **Costs animals** | **Maintenance** | **Operation** | -$ 5.337.149 |
| dic-23 | $ 239.345 | $ 5.162.300 | $ 4.751.504 | $ 57.279 | $ 31.808 | $ 561.053 |
| dic-24 | $ 249.203 | $ 8.193.930 | $ 5.340.295 | $ 47.036 | $ 33.118 | $ 3.022.683 |
| dic-25 | $ 259.467 | $ 8.531.409 | $ 5.560.243 | $ 39.004 | $ 34.482 | $ 3.157.146 |
| dic-26 | $ 270.153 | $ 8.882.789 | $ 5.789.250 | $ 50.991 | $ 35.903 | $ 3.276.798 |
| dic-27 | $ 281.280 | $ 9.248.640 | $ 6.419.245 | $ 82.335 | $ 37.381 | $ 2.990.959 |
| dic-28 | $ 292.865 | $ 9.629.559 | $ 6.275.949 | $ 55.278 | $ 38.921 | $ 3.552.276 |
| dic-29 | $ 304.927 | $ 10.026.168 | $ 6.534.434 | $ 45.838 | $ 40.524 | $ 3.710.299 |
| dic-30 | $ 317.486 | $ 10.439.111 | $ 6.803.565 | $ 59.925 | $ 42.193 | $ 3.850.914 |
| dic-31 | $ 330.562 | $ 10.869.062 | $ 7.083.780 | $ 49.691 | $ 43.931 | $ 4.022.222 |
| dic-32 | $ 344.177 | $ 11.316.721 | $ 7.854.647 | $ 291.054 | $ 45.740 | $ 3.469.457 |
| dic-33 | $ 358.352 | $ 11.782.817 | $ 7.679.309 | $ 53.869 | $ 47.624 | $ 4.360.367 |
| dic-34 | $ 373.111 | $ 12.268.111 | $ 7.995.593 | $ 71.629 | $ 49.585 | $ 4.524.414 |
| dic-35 | $ 388.479 | $ 12.773.392 | $ 8.324.904 | $ 956.980 | $ 51.628 | $ 3.828.358 |
| dic-36 | $ 404.479 | $ 13.299.483 | $ 8.667.778 | $ 76.344 | $ 53.754 | $ 4.906.086 |
| dic-37 | $ 421.138 | $ 13.847.243 | $ 9.611.018 | $ 123.274 | $ 55.968 | $ 4.478.122 |
| dic-38 | $ 438.483 | $ 14.417.563 | $ 9.396.473 | $ 82.763 | $ 58.273 | $ 5.318.537 |
| dic-39 | $ 456.542 | $ 15.011.373 | $ 9.783.481 | $ 68.629 | $ 60.673 | $ 5.555.132 |
| dic-40 | $ 475.346 | $ 15.629.639 | $ 10.186.429 | $ 89.720 | $ 63.172 | $ 5.765.664 |
| dic-41 | $ 494.924 | $ 16.273.370 | $ 10.605.973 | $ 74.399 | $ 65.774 | $ 6.022.148 |
| dic-42 | $ 515.308 | $ 16.943.614 | $ 11.760.129 | $ 435.772 | $ 68.483 | $ 5.194.538 |
| dic-43 | $ 536.532 | $ 17.641.462 | $ 11.497.611 | $ 80.653 | $ 71.303 | $ 6.528.427 |
| dic-44 | $ 558.630 | $ 18.368.053 | $ 11.971.157 | $ 105.440 | $ 74.240 | $ 6.775.845 |
| dic-45 | $ 581.638 | $ 19.124.570 | $ 12.464.208 | $ 87.434 | $ 77.298 | $ 7.077.268 |
| dic-46 | $ 605.593 | $ 19.912.245 | $ 12.977.566 | $ 114.304 | $ 80.482 | $ 7.345.486 |
| dic-47 | $ 630.535 | $ 20.732.361 | $ 14.389.802 | $ 184.568 | $ 83.796 | $ 6.704.731 |
|  |  |  |  |  |  |  |
| **Present value** | $ 6.215.605 | $ 201.756.649 | $ 134.583.769 | $ 2.082.827 | $ 826.035 |  |
| **Total discounted benefits** | $ 207.972.254 |  |  |  |  |  |
| **Total discounted costs** | $ 142.829.781 |  |  |  |  |  |

**Appendix K.** Sensitivity Analysis

Fig. K1 Scores by varying the weight of the economic criteria (weight of technical criteria=0%)

Fig. K2 Scores by varying the weight of the technical criteria (weight of economic criteria=0%)

Fig. K3 Scores by varying the weight of the social criteria (weight of economic criteria=0%)

**References**

Abdulla FA, Al-Shareef AW (2009) SI Roof rainwater harvesting systems for household water supply in Jordan. Desalination 243:195–207. https://doi.org/10.1016/j.desal.2008.05.013

Ávila A (2013) SI Ecotecnia para captación y reciclaje de aguas pluviales en casas de interés social en Pachua, Hidalgo. Universidad Nacional Autónoma de México

CAWST (2009) SI Manual para el filtro de bioarena diseño, construcción, instalación, operación y mantenimiento

Mazurkiewicz K, Jeż-Walkowiak J, Michałkiewicz M (2022) SI Physicochemical and microbiological quality of rainwater harvested in underground retention tanks. Sci Total Environ 814:. https://doi.org/10.1016/j.scitotenv.2021.152701

Nossa Fuentes SC (2017) SI Alternativas de desinfección el agua para el siglo XXI sin cloro. Universidad de Pamplona–Facultad de Ingenierías y Arquitectura.

Posadas A (2015) SI Sistema de cosecha de agua pluvial y reutilización de aguas grises de regadera en vivienda unifamiliar. Tesis de maestría

Ramírez L, Pérez N (2002) SI Uso de filtros lentos para el tratamiento de agua a nivel domiciliario. Ing Hidráulica y Ambient 23:44–50

Unatsabar (2001) SI Guía de diseño para captación del agua de lluvia
